# Effectiveness of a Diabetes-Focused Electronic Discharge Order Set and Postdischarge Nursing Support Among Poorly Controlled Hospitalized Patients: Randomized Controlled Trial

**DOI:** 10.2196/33401

**Published:** 2022-07-26

**Authors:** Audrey White, David Bradley, Elizabeth Buschur, Cara Harris, Jacob LaFleur, Michael Pennell, Adam Soliman, Kathleen Wyne, Kathleen Dungan

**Affiliations:** 1 Internal Medicine University of Virginia Charlottesville, VA United States; 2 Division of Endocrinology, Diabetes & Metabolism The Ohio State University Columbus, OH United States; 3 College of Medicine The Ohio State University Columbus, OH United States; 4 Division of Biostatistics Department of Biostatistics The Ohio State University College of Public Health Columbus, OH United States

**Keywords:** type 2 diabetes, discharge, order set

## Abstract

**Background:**

Although the use of electronic order sets has become standard practice for inpatient diabetes management, there is limited decision support at discharge.

**Objective:**

In this study, we assessed whether an electronic discharge order set (DOS) plus nurse follow-up calls improve discharge orders and postdischarge outcomes among hospitalized patients with type 2 diabetes mellitus.

**Methods:**

This was a randomized, open-label, single center study that compared an electronic DOS and nurse phone calls to enhanced standard care (ESC) in hospitalized insulin-requiring patients with type 2 diabetes mellitus. The primary outcome was change in glycated hemoglobin (HbA_1c_) level at 24 weeks after discharge. The secondary outcomes included the completeness and accuracy of discharge prescriptions related to diabetes.

**Results:**

This study was stopped early because of feasibility concerns related to the long-term follow-up. However, 158 participants were enrolled (DOS: n=82; ESC: n=76), of whom 155 had discharge data. The DOS group had a greater frequency of prescriptions for bolus insulin (78% vs 44%; *P*=.01), needles or syringes (95% vs 63%; *P*=.03), and glucometers (86% vs 36%; *P*<.001). The clarity of the orders was similar. HbA_1c_ data were available for 54 participants in each arm at 12 weeks and for 44 and 45 participants in the DOS and ESC arms, respectively, at 24 weeks. The unadjusted difference in change in HbA_1c_ level (DOS – ESC) was −0.6% (SD 0.4%; *P*=.18) at 12 weeks and −1.1% (SD 0.4%; *P=*.01) at 24 weeks. The adjusted difference in change in HbA_1c_ level was −0.5% (SD 0.4%; *P*=.20) at 12 weeks and −0.7% (SD 0.4%; *P*=.09) at 24 weeks. The achievement of the individualized HbA_1c_ target was greater in the DOS group at 12 weeks but not at 24 weeks.

**Conclusions:**

An intervention that included a DOS plus a postdischarge nurse phone call resulted in more complete discharge prescriptions. The assessment of postdischarge outcomes was limited, owing to the loss of the long-term follow-up, but it suggested a possible benefit in glucose control.

**Trial Registration:**

ClinicalTrials.gov NCT03455985; https://clinicaltrials.gov/ct2/show/NCT03455985

## Introduction

### Scope and Impact of Diabetes

Type 2 diabetes mellitus (T2D) is a major public health problem that is prevalent in 37.3 million US adults and has been steadily increasing [1]. Diabetes is known to lead to considerable morbidity and mortality, with 39% developing chronic kidney disease, 12% reporting severe vision loss, and nearly 290,000 deaths annually [1]. As the prevalence of diabetes increases, complications can occur and hospitalizations are expected to follow. Diabetes is present in at least 25% of hospitalized patients [2], and hospitalizations for hyperglycemic crises have increased over time [3].

Many complications of diabetes are preventable with comprehensive care, including glycemic control [4]. However, despite the increasing availability of numerous therapeutic classes of medications, the proportion of individuals achieving a glycated hemoglobin (HbA_1c_) level of <7% has declined over time [5]. The reasons for this finding are complex and multifactorial, including changes in demographics, practice patterns, health care policy, and the social and economic context [6].

### Challenges With Hospital Transitions of Care

Hospitalization presents an opportunity to identify potentially vulnerable patients with diabetes and to impact their glucose control, but additional system-based barriers may also occur. During hospitalization, expert guidelines generally recommend the discontinuation of preadmission therapies in favor of an insulin regimen that contains basal, prandial, and correction components [7,8]. In addition, patients receiving insulin before admission often undergo an adjustment in dose owing to changes in oral intake or illness-related factors, and the type of insulin may differ owing to restrictions in hospital formularies. In patients on non–insulin-based regimens who do not achieve glycemic goals, intensification of insulin therapy at discharge may be required. These changes in therapy that occur during hospitalization can magnify the treatment gaps during the transition from hospital to home.

### Consequences of Ineffective Diabetes Discharge Procedures

Unfortunately, effective hospital discharge programs for patients with diabetes are understudied [9-11]. In particular, patients who initiate or intensify insulin therapy have the greatest benefit in glycemic control [10,12]. However, these patients are also particularly vulnerable to transitions in care for a variety of reasons, including the complexity of therapy, differences in dosing and administration in the hospital compared with home, inconsistent or inadequate education in the hospital setting, differences in patient and provider expectations, and insufficient resources and access to care [13,14]. Disruption of insulin therapy following hospitalization is associated with higher HbA_1c_ levels after discharge, shorter survival, and increased frequency of readmission and medical costs [15]. Insulin therapy could be interrupted intentionally or more likely via unintentional means, including missing prescriptions or associated supplies, unclear instructions for use, or other barriers, such as cost and coverage issues, medication complexity, low health literacy, and limited access to care.

### Role of Discharge Order Sets

In a Society of Hospital Medicine Survey, only one-fourth of hospitals were supported by written protocols to standardize medication, education, equipment, and follow-up instructions for hospitalized patients with diabetes [16]. Despite being the most frequently used task-specific order set during hospitalization [17], order sets have not been used to guide insulin use at hospital discharge [18]. Preliminary studies at our institution demonstrated that a switch to a new electronic medical record (EMR) platform resulted in an increase in unclear prescriptions for insulin at the time of discharge, in part owing to the use of a free text field in insulin prescriptions [19]. This study assessed whether a diabetes-focused inpatient discharge order set (DOS) with nurse follow-up calls can improve postdischarge outcomes compared with enhanced standard care (ESC) among hospitalized patients with insulin-requiring T2D.

## Methods

### Design and Participants

This was a single center, 24-week randomized open-label parallel group controlled trial. The inclusion criteria were hospitalized patients aged 25 to 75 years with T2D for at least a 3-month duration, an HbA_1c_ level of >8.5% (69 mmol/mol) within 3 months before enrollment, requiring at least 10 units of basal insulin per day while in the hospital, and able to provide informed consent. The age 25 years was chosen to minimize the possibility of inadvertently including type 1 diabetes [20,21], while the age 75 years was chosen to minimize the inclusion of patients who were less likely to discharge home [22]. Participants were required to have access to a phone or electronic messaging post discharge. Exclusion criteria included inmates, pregnancy, inability to consent, or patients with an expected need for skilled nursing facility stay greater than 2 weeks.

Participants were identified through daily screening of inpatient medical and surgical services throughout the institution and were enrolled between January 5, 2018, and April 3, 2020. Permission was obtained from the attending physician of the inpatient service by the study coordinator before approaching the patient in person.

### Sample Size

We estimated a sample size of 111 individuals per group to achieve 80% power to detect a treatment difference of 0.8% in HbA_1c_ levels, adjusting for baseline clinical factors (age, insulin dose at discharge, and whether the patient was new to insulin), assuming 20% attrition, σ=2.2% (SD of HbA_1c_ levels), ρ=.25 (correlation between HbA_1c_ levels at baseline and at 24 weeks), and *R*^2^=0. 5 (squared correlation between baseline factors and the outcome) [23]. However, study enrollment was halted in March 2020 owing to the COVID-19 pandemic crisis and concerns about the feasibility of continuing to enroll and conduct study visits.

### Intervention

Randomization to the DOS or ESC was performed in a 1:1 ratio using a random number generator program within an electronic data capture system (REDCap [Research Electronic Data Capture; Vanderbilt University]) and was stratified by admission insulin therapy.

The DOS was developed with the consultation of a multidisciplinary team that included feedback from Hospital Medicine and diabetes specialists of the hospital. Before the development of the DOS, discharge orders were not specifically tailored to the patient with diabetes. In the DOS, the following orders are accompanied by preselected options with additional cascading options to enhance decision support (Table S1 in Multimedia Appendix 1):

- Diet: there are multiple choices from regular to enteral feeding. The DOS presents 2 separate choices, one for a consistent carbohydrate diet and the other for a flexible carbohydrate diet intended for the patient with carbohydrate counting skills. The goal is to help link the patient’s insulin regimen to their diet.
- Follow-up appointments or referrals: prepopulated choices for primary care, endocrinology, and diabetes education, with prompts to consider outside referrals for patients living outside the catchment area to increase the likelihood of follow-up.
- Medications: for hospitalized patients, neither the preadmission order nor the hospital order for insulin is typically appropriate for a patient at discharge. Moreover, such orders are often complex, and ancillary orders such as pen needles or syringes may be omitted. Thus, insulin options in the DOS are presented via a pick list with linked panels containing a prefilled quantity of pen needles or syringes as appropriate, and default text with decision support that assists the prescriber in choosing the appropriate dose adjustments (eg, basal insulin titration or short or rapid acting correction scale) if indicated.
- Glucose monitoring: these supplies are rarely addressed in admission or discharge orders. The testing supplies in the DOS are bundled (monitor, test strips, and lancets) with default instructions and prefilled quantities, according to the frequency of glucose monitoring.
- Additional supplies: glucagon orders and ketone strips are presented as options with default prescribing instructions.
- Education: additional instructions, including glucose targets and insulin administration, are provided as preselected options.

No modifications were made to the DOS during the study period. The DOS is embedded within the discharge navigator of the EMR (Epic). The DOS also provided instructions to the patients for basal insulin dose self-titration. Default instructions advised patients to increase the dose of insulin glargine 300 U/mL (Gla-300) by 2 units every 4 days for fasting glucose greater than 130 mg/dL, provided no values were less than 80 mg/dL. These instructions could be amended by the discharge team or the primary care provider. Other than Gla-300, no additional prescriptions were pended. In the DOS arm, the primary team was instructed to verify and complete the DOS launched by the study team.

All participants in both treatment groups received a phone call at 2 and 6 weeks following discharge, in which data related to ambulatory and inpatient encounters, glucose monitoring, and insulin use were collected. Basal insulin adherence was defined as >80% of the doses taken in the previous week, and the participants in the DOS received follow-up telephone calls by the study nurse to facilitate ongoing basal insulin dose titration and hospital follow-up. The nurse had a basic understanding of diabetes but was not a certified diabetes care and education specialist. In the ESC group, follow-up telephone calls and visits were conducted on the same schedule as in the ESC group but were conducted by the study coordinator for the purposes of information gathering only. In-person visits at 12 and 24 weeks were conducted by the coordinator in the ESC group and by the nurse with or without the coordinator in the DOS group. During in-person visits, HbA_1c_ and point-of-care glucose levels were collected, in addition to data collected during telephone visits. Patient retention efforts included face-to-face visits with a study investigator before all enrollments to confirm the willingness to complete all study visits, identifying multiple methods of contacting the patient, identifying emergency contacts, and performing study visits during hospitalization when a patient was readmitted.

### Background Therapy and Procedures

All participants received Gla-300 (provided at no cost to the participant) plus additional background therapy (noninsulin and prandial insulin therapies) as part of standard care, as determined by the hospital discharge team. A basic description of Gla-300 was provided to hospital teams using a standard template, recommending that Gla-300 be administered in 1 or 2 injections per day at the same time of day (only pens with 1 unit dosing increments were available). A 1:1 initial dosing conversion from in-hospital administration of glargine 100 U/mL or detemir to outpatient Gla-300 was recommended. The Diabetes Consult Service provided input only when requested by the primary service.

All patients received standard discharge instructions using the EMR, which features medication reconciliation, prescription generation, disease-specific instructions, and follow-up appointments. Hospital discharge was coordinated by the primary team and case manager, who arranged follow-up and any additional needs, such as transportation before discharge. A discharge summary is sent to the primary care provider of the records per routine practice. All patients were instructed to maintain a standardized study diary that recorded glucose levels and insulin dose by the time of day, as well as any hypoglycemic events and associated symptoms.

### Analysis

The primary outcome was the change in HbA_1c_ levels from baseline to 24 weeks post discharge.

Secondary outcomes related to hospital discharge included the proportion of patients with prescriptions for insulin and related supplies, and clear patient instructions (including correct frequency, no jargon or technical terms, correct quantity dispensed, and refills) using the following definitions:

- Jargon: any medical abbreviation or terms (introduced via use of free text fields for bolus insulins during medication reconciliation or prescription generation) that inappropriately appear in patient discharge instructions. Examples include “CIR,” “ICR,” “CF,” “ISF,” “QAC,” “HS,” “SQ,” “Q,” “TID,” “1:50>150,” “SS,” “SSI,” and “subcutaneous.”
- Quantity: the appropriate quantity was determined from the dose or number required for at least a 30-day supply. The frequency of glucose testing was assumed to be 3 or more times per day, because all patients required insulin.
- Refills: present if any refill was provided.
- Bolus error: this refers to omission or incorrect frequency or quantity, lack of refill for a given bolus prescription, or use of jargon or technical terms or abbreviations in the discharge instructions.
- Any error: this refers to any error (such as use of jargon, incorrect frequency, or quantity) in, or omission of any insulin, syringes, pen needles, or testing equipment.
- Carbohydrate counting refers to the adjustment of the bolus insulin dose based on the carbohydrate to insulin ratio.

The study investigator (KD) confirmed after the study coordinators (JL and AS) collected these data. All study staff received training using a standardized slide set and in-person instructions.

Data collection was conducted using REDCap, which features branching logic relative to each discharge order as relevant. Each patient was interviewed at enrollment to determine the supplies needed at the time of discharge. Post discharge, self-reported insulin dosing and hypoglycemia were solicited by the study coordinator.

Secondary outcomes also included HbA_1c_ at 12 weeks; fasting glucose at 12 and 24 weeks; and the proportion of participants achieving an HbA_1c_ level of <7% (53 mmol/mol), an HbA_1c_ level of <6.5% (48 mmol/mol), or an individualized HbA_1c_ target, defined using the Health Care Effectiveness Data and Information Set (HEDIS) criteria [24]. HbA_1c_ levels and health care use data were collected at study visits or extracted from the EMR (when available) for participants with missing data. All secondary outcomes were prespecified, except for hospital readmission, which was considered an exploratory outcome.

All outcomes were assessed according to the group originally assigned. Follow-up data, including primary and secondary outcomes, were analyzed using generalized linear mixed models. Continuous outcomes were analyzed using linear mixed models, assuming an unstructured covariance matrix for residual errors, and binary outcomes were analyzed using logistic regression models containing random subject-specific intercepts. For some binary outcomes, we could not fit mixed models because of small cell counts. In these cases, data were analyzed cross-sectionally using separate logistic regression models fitted to the data at each time point or Fisher’s exact test, depending on the number of events. All models were adjusted for potential confounders, which we defined as any factors measured at baseline related to the outcome (*P*<.10) that differed by a meaningful amount across treatment arms (difference in proportions of 10% or more, difference in means of 0.5 SDs or more) at any follow-up visit (Table S2 in Multimedia Appendix 1). In the primary analysis (change in HbA_1c_), we also adjusted for risk factors identified a priori (age, insulin dose at discharge, and whether the patient was new to insulin) to increase the precision of our treatment effect estimate. In secondary analyses, the Holm method [24] was used to account for multiple comparisons performed at 2, 6, 12, and 24 weeks [25]. Differences in the components ordered at discharge were analyzed using the Fisher Exact Test. Analyses were performed using SAS (version 9.4) and JMP 13.1 (SAS Inc).

### Ethics Approval

This study was approved by the Ohio State University Institutional Review Board (2017H0354), and all patients provided informed consent.

## Results

### Overview

A total of 158 patients signed a consent form (Multimedia Appendix 2). Three patients did not receive the study intervention owing to a withdrawal of consent (2 patients, 1 in each study arm) or no longer qualified owing to a switch to U500 insulin before discharge (1 patient who was randomized to the DOS arm did not receive the intervention (Gla-300) because the patient was treated with U500 insulin, a regimen that does not require basal insulin). The participants had a mean age of 52 (SD 10.2) years, a median duration of diabetes of 11 years, and 81.9% (127/155) were on insulin therapy before hospital admission. Baseline characteristics of each group are presented in Table 1. The treatment groups were similar except for an imbalance in diabetes duration, marital status, and neuropathy.

**Table 1:** Patient characteristics (number of patients overall: N=158; patients in the enhanced standard care [ESC] arm: n=76; patients in the discharge order set [DOS] arm: n=82).

			Overall		ESC		DOS
Age (years), mean (SD)			51.7 (10.2)		51.4 (10.5)		52 (10.1)
Male, n (%)			68 (43)		33 (43.3)		35 (42.7)
White,^a,b^ n (%)			74 (46.8)		34 (44.7)		40 (48.8)
Hispanic, n (%)			3 (1.9)		1 (1.3)		2 (2.4)
Diabetes duration (years), median (IQR)			11 (7-20)		14 (7-20)		10 (6-15)
BMI (kg/m^2^), mean (SD)			38.2 (9.5)		38.1 (8.7)		38.4 (10.1)
**Past medical history, n (%)**							
	Hypertension	134 (84.8)		64 (84.2)		70 (85.4)	
	Hyperlipidemia	98 (62)		45 (59.2)		53 (64.6)	
	Coronary artery disease	44 (27.9)		18 (23.7)		26 (31.7)	
	Heart failure	37 (23.4)		17 (22.4)		20 (24.4)	
	Cerebrovascular disease	21 (13.3)		12 (15.8)		9 (11)	
	Peripheral vascular disease	14 (8.9)		5 (6.6)		9 (11)	
	Retinopathy	28 (17.7)		16 (21.1)		12 (14.6)	
	Nephropathy	39 (24.7)		19 (25)		20 (24.4)	
	Neuropathy	81 (51.3)		45 (59.2)		36 (43.9)	
**Estimated glomerular filtration rate (mL/min/1.73 m^2^), mean (SD)**							
	>60	109 (69)		51 (67.1)		58 (70.7)	
	30-60	39 (24.7)		21 (27.6)		18 (22)	
	<30	107 (6.3)		4 (5.3)		6 (7.43)	
Charlson Comorbidity Index (total score), median (IQR)			3 (2-5)		3 (2-4.75)		3 (2-5)
**Education, n (%)**							
	Less than high school	15 (9.5)		10 (13.2)		5 (6.1)	
	High school or equivalent	118 (74.7)		55 (72.4)		63 (76.8)	
	Bachelor’s degree	25 (15.8)		11 (14.5)		14 (17.1)	
**Marital status, n (%)**							
	Single, never married	46 (29.1)		22 (30)		24 (29.3)	
	Married or domestic partnership	66 (41.8)		25 (32.9)		41 (50)	
	Divorced, separated, or widowed	46 (29.1)		29 (38.2)		17 (20.7)	
**Work status, n (%)**							
	Employed	63 (39.9)		33 (43.4)		30 (36.6)	
	Unemployed	23 (14.6)		11 (14.5)		12 (14.6)	
	Retired	21 (13.3)		10 (13.2)		11 (13.4)	
	Unable to work	51 (32.3)		22 (29)		29 (35.4)	
**Home ownership, n (%)**							
	Own	58 (36.7)		28 (36.8)		30 (36.6)	
	Other	100 (63.3)		48 (63.1)		52 (63.4)	
**Insurance, n (%)**							
	None	11 (7)		7 (9.2)		4 (4.9)	
	Private	52 (32.9)		22 (29.0)		30 (36.7)	
	Medicare	35 (22.1)		18 (23.7)		17 (20.7)	
	Medicaid	60 (38)		29 (38.2)		31 (37.8)	
**Primary reason for admission**, **n (%)**							
	Cardiovascular	40 (25.3)		21 (27.6)		19 (23.2)	
	Gastrointestinal	16 (10.1)		8 (10.5)		8 (9.8)	
	Infectious disease	28 (17.7)		12 (15.8)		16 (19.5)	
	Other	74 (46.8)		35 (46.1)		39 (47.6)	
**Admission service**, **n (%)**							
	General medicine	33 (20.9)		16 (21.1)		17 (20.7)	
	Family medicine	5 (3.2)		3 (4)		2 (2.4)	
	Cardiology	23 (14.5)		15 (19.7)		8 (9.8)	
	Surgery	6 (3.8)		2 (2.6)		4 (4.9)	
Admission severe hyperglycemia,^c^ n (%)			19 (12.1)		8 (10.5)		11 (13.6)
Hospital length of stay (days), median (IQR)			5 (3-8)		5 (3-8)		5 (3-8)
Diabetes consult, n (%)			62 (39.2)		26 (34.2)		36 (43.9)
Education consult, n (%)			29 (18.4)		11 (14.5)		18 (22)
**Admission diabetes medications, n (%)**							
	Any insulin	127 (80.9)		63 (82.9)		64 (79)	
	Basal insulin	126 (80)		64 (84.2)		62 (76.5)	
	Premix insulin	1 (0.64)		1 (1.3)		0 (0)	
	Bolus insulin	82 (52.2)		40 (52.6)		42 (51.9)	
	Metformin	53 (33.5)		25 (32.9)		28 (34.2)	
	Sulfonylurea or glinide	12 (7.6)		7 (9.2)		5 (6.1)	
	SGLT2^d^ inhibitor	11 (7)		4 (5.3)		7 (8.5)	
	DPP-4^e^ inhibitor	6 (3.8)		4 (5.3)		2 (2.4)	
	GLP-1^f^ receptor agonist	26 (16.5)		14 (18.4)		12 (14.6)	
	Other	1 (0.63)		0 (0)		1 (1.2)	
**Other admission medications, n (%)**							
	Statin	120 (76)		57 (75)		63 (76.8)	
	ACEI^g^ or ARB^h^	80 (50.6)		41 (54)		39 (47.6)	
	β-blocker	73 (46.2)		37 (46.7)		36 (43.9)	
	Glucocorticoids	5 (3.2)		1 (1.3)		4 (4.9)	
	Aspirin	84 (53.2)		42 (55.3)		42 (51.2)	
**Discharge diabetes medications**							
	Total insulin dose (unit), median (IQR)	68 (42-115)		74 (43-116)		68 (37.8-112.5)	
	Total insulin dose (unit/kg/day), median (IQR)	0.61 (0.38-1.03)		0.59 (0.39-1.03)		0.69 (0.39-1.03)	
	Basal insulin	41 (30, 75)		50 (30, 74)		41 (30, 78.7)	
	Bolus insulin	123 (79)		63 (79)		60 (80)	
	Metformin, n (%)	56 (36.1)		26 (34.7)		30 (37.5)	
	Sulfonylurea or glinide, n (%)	6 (3.8)		3 (4)		3 (3.8)	
	SGLT2-inhibitor, n (%)	5 (3.2)		5 (6.7)		0 (0)	
	DPP-4 inhibitor, n (%)	10 (6.5)		6 (8)		4 (5)	
	GLP-1 receptor agonist, n (%)	24 (15.5)		14 (18.7)		10 (12.5)	
Diabetes empowerment scale [26], median (IQR)			4.4 (4-4.8)		4.4 (4-4.8)		4.3 (3.9-4.8)
Functional health literacy [27], median (IQR)			4 (2-6)		4 (2-6)		5 (3-6)
Multidimensional scale of perceived social support [28], median (IQR)			6 (4.9-6.8)		6.1 (5-6.8)		5.9 (4.7-6.8)

^a^Race was categorized as White (46.5%), Black (52.3%), Asian (0.65%), or other (0.65%).

^b^Chi-square analysis could not be performed owing to insufficient cell count.

^c^Admission for diabetic ketoacidosis, nonketotic hyperglycemic hyperosmolar state, or diabetes as the primary indication for admission.

^d^SGLT2: sodium-glucose cotransporter-2.

^e^DPP-4: dipeptidyl peptidase-4.

^f^GLP-1: glucagon-like peptide-1.

^g^ACEI: angiotensin-converting enzyme inhibitor-1.

^h^ARB: angiotensin receptor blocker.

### Discharge Orders

Discharge data were available in 75 participants in the ESC arm and 80 participants in the DOS arm. Analysis of discharge orders is shown in Table 2. Among patients reporting an insufficient supply, those in the DOS arm were more likely to receive prescriptions for bolus insulin (21/27, 78%, vs 12/27, 44%; *P*=.01), needles and syringes (18/19, 95%, vs 15/24, 63%; *P*=.03), and glucometers (24/28, 86%, vs 9/25, 36%; *P*<.001). During hospitalization, most participants reported sufficient home supplies of bolus insulin (78/119, 66%), lancets (80/155, 52%), and a glucometer (102/155, 66%). However, needles and syringes were sufficient in only 7% (3/46) of patients, and test strips were sufficient in only 43% (66/155). No continuous or intermittently scanned glucose monitors were used. Overall, the errors in discharge orders were similar between the arms (44/80, 55%, in DOS vs 51/75, 68%, in ESC). Among patients in need of a bolus insulin prescription, errors occurred in 53% (9/17) of the DOS group and 79% (19/24) of the ESC group (*P*=.10). Medical jargon was present in 29% (5/17) of the DOS group and 38% (9/24) of the ESC group (*P*=.74). Patients in the DOS arm were more likely to receive needles or syringes in the correct quantity (17/19, 89%, vs 14/24, 58%; *P*=.04). The number of participants reporting a need for test strips or lancets who received both was 79% (27/34) in the DOS group and 59% (23/46) in the standard of care group (*P*=.08). For those who were prescribed bolus insulin and who reported needing supplies at baseline, 94.1% (16/17) in the DOS and 57.1% (12/21) in the standard of care received both prescriptions (*P*=.01).

**Table 2:** Discharge order set.

		Overall		Enhanced standard care		Discharge order set		*P* value^a^
		n (%)	N	n (%)	N	n (%)	N	
**Bolus insulin**								
	Bolus insulin at discharge	123 (79.4)	155	60 (80)	75	63 (78)	80	.99
	Home supply sufficient	78 (65.6)	119	33 (58)	57	45 (73)	62	.13
	Prescription provided^b^	33 (61.1)	54	12 (44)	27	21 (78)	27	*.01*
	Jargon present^b^	14 (34.1)	41	9 (38)	24	5 (29)	17	.74
	Any bolus error present^b,c^	28 (68.3)	41	19 (79)	24	9 (53)	24	.10
	Carbohydrate counting^d^	8 (5.3)	152	5 (7)	73	3 (4)	79	.48
**Basal insulin**								
	Correct basal insulin ordered	100 (64.9)	155	44 (59)	75	56 (71)	80	.13
**Needles and syringes**								
	Home supply sufficient	3 (6.5)	46	2 (8)	26	1 (5)	20	.99
	Prescription provided^b^	33 (76.7)	43	15 (63)	24	18 (95)	19	*.03*
	Correct quantity^b^	31 (72.1)	43	14 (58)	24	17 (89)	19	*.04*
**Glucometer**								
	Glucometer at home	102 (65.8)	155	50 (67)	75	52 (65)	80	.87
	Prescription provided^b^	33 (62.3)	53	9 (36)	25	24 (86)	28	*<.001*
**Test strips**								
	Home supply sufficient	66 (42.6)	155	32 (43)	75	34 (43)	80	.99
	Prescription provided^b^	59 (66.3)	89	25 (58)	43	34 (74)	46	.12
	Correct quantity^b^	59 (66.3)	89	25 (58)	43	34 (74)	46	.12
**Lancets**								
	Home supply sufficient	80 (51.6)	155	35 (47)	75	45 (56)	80	.26
	Prescription provided^b^	48 (64.9)	74	21 (54)	39	27 (77)	35	.05
	Correct quantity^b^	48 (64.9)	74	21 (54)	39	27 (77)	35	.05
Any error		95 (61.3)	155	51 (68)	75	44 (55)	80	.10

^a^*P* values with statistical significance are italicized.

^b^Among patients with insufficient supply and in need of a prescription.

^c^Bolus error refers to any error in frequency or quantity or the use of jargon, technical terms, or abbreviations in the discharge instructions.

^d^Adjusting bolus insulin dose based on the carbohydrate to insulin ratio.

### HbA_1c_ and Glucose

HbA_1c_ and glucose measurements are shown in Table 3. HbA_1c_ was available in 54 participants in each arm at 12 weeks, and 44 and 45 participants in the DOS and ESC arms, respectively, at 24 weeks. The remaining participants were lost to follow-up. There was no difference in baseline characteristics according to HbA_1c_ availability at weeks 12 or 24 (Table S3 in Multimedia Appendix 1). The change in HbA_1c_ at 12 weeks was −2% (SD 0.3%; 22, SD 3.3 mmol/mol) vs −1.4% (SD 0.3%; 15, SD 3.3 mmol/mol) at 12 weeks and −2.1% (SD 0.3%; 23, SD 3.3 mmol/mol) vs −1.0% (SD 0.3%; 11, SD 3.3 mmol/mol) at 24 weeks in the DOS and ESC arms, respectively. The differences between the groups were not significant after adjustment for age, neuropathy, total daily insulin dose, and reason for hospitalization. The proportions of participants achieving a target HbA_1c_ level of <7% (53 mmol/mol) or <6.5% (48 mmol/mol) were similar. Participants in the DOS arm were more likely to achieve an HbA_1c_ level below their HEDIS target at 12 weeks (22/54, 41%, vs 9/54, 17%; odds ratio [OR] 3.29, 95% CI 1.32-8.13; *P*=.01); although this association did not persist for 24 weeks (16/45, 36%, vs 9/45, 20%; OR 2.10, 95% CI 0.80-5.55; *P*=.13). Fasting glucose levels were similar between the groups at the 12- and 24-week study visits.

**Table 3:** Follow-up glucose and glycated hemoglobin (HbA_1c_) data.

				12 weeks							24 weeks				
				Enhanced standard care		Discharge order set		*P* value^a^		Enhanced standard care			Discharge order set		*P* value^a^
**HbA_1c_^b^**															
	Number at discharge			n=73		n=79		N/A^c^		N/A			N/A		N/A
	Discharge HbA_1c_ (%), median (IQR)			10.9 (9.8-12)		10.7 (9.5-11.9)		N/A		N/A			N/A		N/A
	Discharge HbA_1c_ (mmol/mol), median (IQR)			96 (67-108)		93 (80-107)		N/A		N/A			N/A		N/A
	Number at follow-up			n=54		n=54		N/A		n=45			n=44		N/A
	Observed data (%), median (IQR)			8.9 (8.1-11.3)		8.7 (7.2-10.1)		N/A		9.5 (7.8-12.2)			8.3 (7.5-10)		N/A
	Observed data (mmol/mol), median (IQR)			74 (65-100)		72 (55-87)		N/A		80 (62-110)			67 (58-86)		N/A
	Change from baseline (%),^d^ mean (SE)			−1.4 (0.3)		−2 (0.3)		N/A		−1.0 (0.3)			−2.1 (0.3)		N/A
	Change from baseline (mmol/mol),^d^ mean (SE)			15 (3)		22 (3)		N/A		11 (3)			23 (3)		N/A
	Difference in change,^d,e^ mean (SE)			Reference		−0.6 (0.4)		.18		Reference			−1.1 (0.4)		.01
	Adjusted difference in change,^d,e,f^ mean (SE)			Reference		−0.5 (0.4)		.20		Reference			−0.7 (0.4)		.09
	HbA_1c_ <7% (53 mmol/mol),^g^ n (%)			2 (3.7)		7 (13.0)		.16		2 (4.4)			3 (6.8)		.68
	HbA_1c_ <6.5% (48 mmol/mol),^g^ n (%)			1 (1.9)		4 (7.4)		.36		0 (0)			3 (6.8)		.12
	HbA_1c_ <HEDIS^h^ target, n (%)			9 (16.7)		22 (40.7)				9 (20)			16 (36.4)		
	HbA_1c_ <HEDIS target,^i^ OR^j^ (95% CI)			Reference		3.29 (1.32-8.13)		.01		Reference			2.1 (0.8-5.55)		.13
**Point-of-care glucose**															
	**Fasting only**			n=27		n=27		N/A		n=21			n=20		N/A
		Observed data, median (IQR)	212 (149-258)		166 (142-239)		N/A		209 (129.5-234)			152.5 (127.3-247.3)		N/A	
		Adjusted difference,^e^ mean (SE)	Reference		−18 (23)		.44		Reference			−26.5 (30.3)		.39	
	**Any**			n=40		n=45		N/A		n=33			n=33		N/A
		Observed data, median (IQR)	209.5 (133.8-258)		179 (150.5-144.5)		N/A		209 (136.5-295)			161 (134-230)		N/A	
		Adjusted difference^k^	Reference		−23.9 (20.8)		.25		Reference			−30.4 (21.9)		.17	

^a^Estimated using a linear mixed model.

^b^Data for follow-up HbA_1c_ levels were collected at study visits and, when possible, extracted from the electronic medical records. All other data were obtained during the study visits. One death occurred at 24 weeks in the DOS group.

^c^N/A: not applicable.

^d^Change from baseline in discharge order set; change from baseline in enhanced standard care.

^e^Adjusted for age, work status, insurance, and functional health literacy scores. Two participants were excluded from the analysis because of missing functional health literacy scores.

^f^Adjusted for age, neuropathy, total daily insulin dose, insulin before admission, reason for hospitalization, and metformin use at discharge.

^g^Mixed models could not be fit owing to small cell sizes; Fisher Exact Tests were performed instead.

^h^HEDIS: Health Care Effectiveness Data and Information Set (target is <8% if age ≥65 years or known history of ischemic vascular disease, heart failure, advanced kidney disease [estimated glomerular filtration rate of <30 mL/min/1.73 m^2^], dementia, proliferative retinopathy or blindness, advanced neuropathy [history of ulcer or amputation], or history of severe hypoglycemia; otherwise, goal is <7%).

^i^Estimate (95% CI). From separate logistic regression models fitted to data at each time point, odds ratios adjusted for baseline HbA_1c_ but not for confounders, owing to small cell counts.

^j^OR: odds ratio.

^k^Adjusted for marital status, insurance, and bolus insulin use at admission.

### Health Care Use

Readmissions within 30 days (exploratory outcome) occurred in 17 (13%) of the participants. Among all participants, primary care follow-up was 55%, 74%, and 87% at 2, 6, and 12 weeks while endocrinology visits occurred in 23%, 27%, and 52% of participants at 2, 6, and 12 weeks, respectively. Emergency department visits, readmission rates, primary care, and endocrinology follow-up visits were similar between the groups (Table S4 in Multimedia Appendix 1).

### Diabetes Medications and Hypoglycemia

The basal insulin dose and initiation of glucose-lowering medications were similar between the groups at follow-up (Tables S4 and S5 in Multimedia Appendix 1). At the 2- and 24-week follow-ups, changes in basal insulin dose were similar. However, patients in the DOS arm were significantly more likely to have an increase in basal insulin dose at 12 weeks (25/45, 53%, vs 8/38, 21%; OR 4.70, 95% CI 1.63-13.52), and the difference at 6 weeks was marginally significant (16/34, 49%, vs 8/9, 23%; OR 3.53, 95% CI 1.18-10.62). Patients in the DOS group were also more likely to have a *decrease* in basal insulin dose at 12 weeks (13/45, 28%, vs 1/38, 3%; *P*=.009), but not at other time points (Table S4 in Multimedia Appendix 1). Basal insulin adherence was similar at all follow-up time points (Table S4 in Multimedia Appendix 1). Hypoglycemic events were similar between the groups (Table S4 in Multimedia Appendix 1).

## Discussion

### Principal Findings

In this study, an insulin-specific DOS was developed to address barriers to prescribing insulin and to improve hospital transition of care among persons with T2D. The DOS resulted in improvements in prescriptions for bolus insulin, needles, and testing supplies, but did not improve order clarity. Follow-up data were available for a subset of patients for whom there were favorable trends in adjusted HbA_1c_ levels, despite early discontinuation of study recruitment. These data from the first study to implement a dedicated DOS among hospitalized patients with T2D requiring insulin provide important insights for optimizing hospital diabetes discharge programs.

### Discharge Orders

Medication reconciliation is a cognitively demanding task, particularly when insulin is involved. Despite the readily available electronic medication reconciliation functionality, there is still an opportunity to improve discharge orders for insulin. In this study, a DOS improved the frequency of missing prescriptions for insulin and glucose monitoring supplies. This is of critical importance, because disruption of insulin therapy is a known predictor of hospital readmission, higher HbA_1c_ levels, and increased costs [15]. Moreover, the omission of preadmission diabetes therapies at discharge is associated with higher readmission and mortality rates [15,29,30]. These findings are novel in that proposed interventions to date have included individual provider education, traditional quality improvement initiatives, or hiring pharmacists rather than enhancing electronic decision support [31-34]. Although not an integral component of the DOS studied here, we observed no significant change in the proportion of patients who were prescribed metformin and other noninsulin therapies from admission to discharge. Future iterations should also consider the optimization of noninsulin therapies, particularly to reduce cardiorenal risk [34].

However, there are opportunities to improve the clarity of insulin instructions in the EMR. Dosing fields are typically inadequate, resulting in the need to use free text fields or provide discharge instructions via a separate workflow that is external to the electronic medication reconciliation process [11]. Additional customization could include fields that account for oral intake, glucose level, or time of day, guide patient self-titration, or calculate the quantity dispensed. In particular, decision support could provide guidance for converting flexible meal dosing (the standard practice at the study institution) to fixed meal dosing, which is more appropriate for many patients. Additional benefits could be achieved by implementing a remote monitoring program postdischarge and device integration (smart insulin pen and glucose monitor) within the EMR. Ironically, one artifact may have been introduced by the multifaceted intervention itself, which required a switch in basal insulin at discharge, often late in hospitalization and possibly after other discharge orders were populated. Following the closure of the study and after obtaining feedback from the institution’s multidisciplinary inpatient Glucose Management Committee, additional revisions to the DOS were made, including presenting pens as the preferred choice and adding concentrated and premixed insulin orders.

### Follow-up Data

In this study, HbA_1c_ reduction was evident in both groups, likely owing to the provision of insulin therapy (at least, in part). The individualized HEDIS goal was reached by more patients in the DOS group at 3 months, but there was a waning of effect from 3 to 6 months as the intervention intensity decreased. This phenomenon has been previously described and underscores the need for ongoing high frequency care [10]. The HbA_1c_ reduction was similar to or somewhat smaller in magnitude compared with other prospective nonrandomized studies of recently hospitalized patients [35,36].

A greater proportion of participants had an increase in the basal insulin dose in the DOS group at 6 and 12 weeks but not at 2 weeks, compared with the ESC group. This emphasizes the utility of early hospital follow-ups to review any prescription needs, assess the patient’s understanding of the diabetes regimen, obtain a history of hyperglycemia or hypoglycemia, and remind patients to perform self-titration where relevant. Moreover, early visits could address the need for prandial insulin (to avoid overreliance on basal insulin) if not already prescribed and noninsulin therapies. At the 12-week time point, it is important to establish a plan for continued frequent contact (such as monthly visits by a pharmacist or nurse) to maintain early success.

Despite a favorable trend in HbA_1c_ levels, hypoglycemia was similar in both the groups. While the total daily dose of insulin was reasonable at 0.61 unit/kg, patients tended to have basal-heavy regimens and might have benefited from a dose reduction at discharge to further reduce the frequency of hypoglycemia [36].

While the study population was generalizable owing to the broad inclusion criteria, the limited follow-up data greatly impacted the ability to assess the external validity of the postdischarge component of the intervention. This study was not designed to address many barriers to successful transitions of care, including clinical inertia, as well as patient-specific factors such as mental health, physical disability, literacy, financial hardship, social factors, and lack of transportation [14,37]. While formal diabetes education is of tremendous value in helping prepare patients for hospital discharge [12,38], it is not widely available or reimbursable in the hospital setting. Comprehensive models that incorporate bedside nurses, dietitians, care managers, and pharmacists, possibly in combination with video or web-based education resources with timely feedback may help to bridge the gap in education [12,38,39]. Specially trained navigators, caseworkers, or community health workers can help address other barriers. Finally, multilevel interventions should incorporate frequent contact, target the highest-risk patients, and span multiple domains of care [40,41]. Access to and quality of care should improve as telehealth visits and remote glucose monitoring become mainstream.

### Limitations

As noted in the previous paragraph, the limitations of the study relate to loss to follow-up, even within the context of specifically dedicated study staff and other enhancements. The COVID-19 pandemic presented the largest barrier to carrying out study procedures, as dropout was more common among patients who were enrolled in the 6 months before the start of the pandemic. Unfortunately, we were able to address this issue only partially with telehealth or minimal contact strategies; this highlights the overall vulnerability of our patient population. Furthermore, it is unknown whether similar results would be achieved in other populations (type 1 diabetes, noninsulin requiring, and broader age range). As with other multicomponent interventions, it is difficult to discern which components of the discharge instruction or nursing support were the primary determinants of success. Finally, while the DOS increased the completeness of diabetes medications at discharge, it was launched by the study team to understand its utility under optimal use. Thus, an additional study of the usability, acceptability, and implementation of the DOS is needed. For example, it would have been useful to quantify the time saved through the use of the order set owing to the presence of prefilled fields and decision support.

### Conclusions

An intervention that included electronic DOS plus postdischarge nurse phone calls resulted in more complete discharge prescriptions for insulin and related supplies. However, there is an opportunity to improve the clarity of the instructions. Post discharge HbA_1c_ levels showed favorable trends, but interpretation of data is limited owing to loss of follow-up amid COVID-19 pandemic restrictions. More intensive interventions are needed to optimize postdischarge care.

## Appendix

Multimedia Appendix 1 Supplemental data.

Multimedia Appendix 2 CONSORT (Consolidated Standards of Reporting Trials) diagram (patient flow diagram).

Multimedia Appendix 3 CONSORT-eHEALTH checklist (V 1.6.1).

## Abbreviations

DOS: discharge order set
EMR: electronic medical record
ESC: enhanced standard care
Gla-300: insulin glargine 300 U/mL
HbA_1c_: glycated hemoglobin
HEDIS: Health Care Effectiveness Data and Information Set
OR: odds ratio
REDCap: Research Electronic Data Capture
T2D: type 2 diabetes mellitus
