# Supplementary material for: Effectiveness of a Diabetes-Focused Electronic Discharge Order Set and Postdischarge Nursing Support Among Poorly Controlled Hospitalized Patients: Randomized Controlled Trial
Source: JMIR Diabetes. 2022 Jul 26;7(3):e33401. doi: 10.2196/33401 (PMC9364166; doi:10.2196/33401)
Supplement: Multimedia Appendix 1 [file diabetes_v7i3e33401_app1.docx]

| **Supplementary Table 1. Diabetes Order Set (DOS)**  **NOTE TO ORDERING CLINICIAN:**  **Orders in this OrderSet are built to improve accuracy and appropriateness of Insulin and Supply Orders at Discharge. (May reference patient's HOME Insulin orders and/or Inpatient Insulin Orders and/or any END/Diabetes Consult.)  If selecting an insulin order from this order set, be sure to note that existing inpatient insulin orders must be marked to discontinue at discharge to avoid confusion for the patient.** | | |
| --- | --- | --- |
| **Orders without DOS** | **Orders with DOS** | **Additional Drop-down Choices** |
| **Diet** | | |
| Multiple choices, no default | *Note: Most patients will require consistent carbohydrate diet* | |
|  | - Consistent carbohydrate diet *Note:* *appropriate for most patients, including those discharging to skilled nursing* | - *45 grams per meal* - *60 grams per meal* - *Patient specific* |
|  | - Flexible carbohydrate diet *Note: carbohydrate counting method, only if patient using prior to hospitalization or has demonstrated competency* |  |
| **Follow-up and Referrals** | | |
| No default | - Primary Care Provider | *[see below]* |
|  | - Endocrinologist *Note: If patient does not live near ***, please locate an Endocrinologist nearest to their home. The phone number for OSU Endocrinology is **** | - - Call for an appointment with your *** for 2 weeks after discharge   - Call for an appointment with your *** as directed   - Follow-up appointment arranged with *** on [date] |
|  | - Diabetes Education *Note: If patient does not live near ***, please locate a diabetes educator nearest to their home. The phone number for OSU Endocrinology is **** | - - Survival skills   - General diabetes education   - Outside of *** |
|  | - Bring a blood sugar log (record of your glucose readings), medications and glucose meter with you. |  |
| **Notify Physician** | | |
| No Default | - Call your healthcare provider if you are having recurrent low sugars (less than 70 mg/dl) more than 2 days in a row or if you have any severe low sugars requiring the assistance of someone else to treat. |  |
|  | - Call your healthcare provider if you have recurrent high (above 250 mg/dl) blood sugars for more than 3 days in a row, or if you have glucose readings over 250 mg/dl with new symptoms such as nausea, vomiting, or dizziness. |  |
| **Patient Instructions** | | |
| No default | - Your target glucose is *** mg/dl fasting and under *** mg/dl nonfasting   *<Note: Typical goals for many are 80-130 mg/dl in the morning (fasting) and <180 mg/dl throughout the rest of the day>* |  |
|  | Communication for Nursing Care   - Insulin naïve: Discharge with insulin, How to take your Insulin - Previous insulin user: Discharge with insulin | *[Attachment]* |
| **Medications** | | |
| Order reconciliation includes pre-admission medication and inpatient orders. | **Diabetes Medication and Supply**: *Note: U100 Insulin vials contain 1000 units, U100 pens contain 300 unit/pen, glargine U300 contains 450 unit/pen, degludec U200 contains 600 unit/pen, lispro U200 contains 600 unit/pen. [each prescription option is paired with U100 0.3 or 0.5 ML syringe or pen needles as appropriate]* | |
|  | - **Basal Insulin**    - Glargine U100 Solostar pen and pen needles   - Glargine U300 Solostar pen and pen needles   - Detemir U100 Flextouch pen and pen needles   - Degludec U100 Flextouch pen and pen needles   - Degludec U200 Flextouch pen and pen needles   - Glargine U100 vial and syringes   - Detemir U100 vial and syringes   - NPH U100 vial and syringes | ***[Example Drop-down Menu:]***   - ***Insulin Glargine 300 unit/ml pen***   **** units every day.*   - *Increase *** unit every 4 days until AM (fasting) glucose is under *** mg/dl, provided that you have no glucoses under 80 mg/dl, do not go above *** unit per day.* - *[Cardiac surgery patients]: Reduce dose by 2 unit every day that you wake up with a glucose less than 100 mg/dl or if you have any glucose levels under 80 mg/dl.* - *No titration*   *Dispense: 3 prefilled pens; Refills: 1*   - ***Pen needle 31 G x 4 mm***   *For use with insulin once daily*  *Dispense: 50; Refills: 1* |
|  | - **Set Meal Insulin Dose**: *appropriate for most patients using prandial insulin including those discharged to skilled nursing.*   - Aspart U100 Flextouch pen and pen needles   - Glulisine U100 Solostar pen and pen needles   - Lispro U100 Kwikpen and pen needles   - Lispro U200 Kwikpen and pen needles   - Aspart U100 vial and syringes   - Lispro U100 vial and syringes | ***[Example Drop-down Menu:]***   - ***Insulin Glulisine 100 Unit/ML Pen-injector***   **** units SQ QAC.*   - *High dose correction* - *Standard correction*  \| *If your glucose is this* \| *Add this much insulin to your mealtime dose* \| \| --- \| --- \| \| *150-199*  *200-249*  *250-299*  *300-349*  *350-400*  *Over 400* \| *1*  *2*  *3*  *4*  *5*  *6* \|  - *Low dose correction*   *Dispense: 3 prefilled pens; Refills: 1*   - ***Pen needle 31 G x 4 mm***   *For use with insulin 4 times daily*  *Dispense: 150; Refills: 1* |
|  | - **Flexible Meal Insulin Dose**: *appropriate ONLY if patient using prior to hospitalization, discharging to Dodd Hall or demonstrates competency*   - Aspart U100 Flextouch pen and pen needles   - Glulisine U100 Solostar pen and pen needles   - Lispro U100 Kwikpen and pen needles   - Lispro U200 Kwikpen and pen needles   - Aspart U100 vial and syringes   - Lispro U100 vial and syringes | ***[Example Drop-down Menu:]***   - ***Insulin Glulisine 100 Unit/ML Pen-injector***   *1 unit for every *** grams of carbohydrate SQ QAC.*   - *High dose correction* - *Standard correction*  \| *If your glucose is this* \| *Add this much insulin to your mealtime dose* \| \| --- \| --- \| \| *150-199*  *200-249*  *250-299*  *300-349*  *350-400*  *Over 400* \| *1*  *2*  *3*  *4*  *5*  *6* \|  - *Low dose correction*   *Dispense: 3 prefilled pens; Refills: 1*   - ***Pen needle 31 G x 4 mm***   *For use with insulin 4 times daily*  *Dispense: 150; Refills: 1* |
| **Glucose Monitoring Supplies and Other Orders** | | |
| Order reconciliation includes pre-admission medication and inpatient orders. | **Glucose Monitoring**   - Once per day (non-insulin requiring patients) - 4 times per day before meals and at bedtime - 6 times per day (before and 2 hours after meals) | ***[Example Drop-down Menu:]***   - **Glucose monitor** *(if patient does not have one at home)*   *Dispense: 1; Refills: 0*   - **Glucose test strips**   *Testing 4 times per day, ICD-10: ****  *Dispense 150; Refills: 1*   - **Lancets**   *Testing 4 times per day, ICD-10: ****  *Dispense 150; Refills: 1*   - **Alcohol wipes**   *Testing 4 times per day, ICD-10: ****  *Dispense 150; Refills: 1* |
| **No default** | - **Ketostix** **strips** (*for Type 1 DM or history of DKA*)   *Test urine prn glucose >400 mg/dl or >250 mg/dl with nausea, vomiting or other symptoms of DKA*  *Dispense 50; Refills: 0* |  |
| **No default** | - **Glucagon emergency kit** (*for Type 1 DM or history of severe hypoglycemia or hypoglycemia unawareness*)   *1 mg SQ prn severe hypoglycemia*  *Dispense 1; Refills: 0* |  |

**Supplementary Table 2. Confounders adjusted for in analysis of follow-up data.**

| **Outcome** | **Confounders** |
| --- | --- |
| Endocrinology Follow-up | education, insurance, diabetes duration, GLP-1 receptor agonist use at admission, |
| Readmissions | admission service, diabetes consult, cerebrovascular disease, peripheral vascular disease |
| Change in total daily insulin dose (units) | BMI, diabetes duration, Diabetes Empowerment Scale |
| Change in Basal Insulin Dose (units) | education, BMI, diabetes duration, Diabetes Empowerment Scale, GLP-1 receptor agonist use at discharge, metformin use at admission, cerebrovascular disease |
| Increase in Basal Insulin Dose | None |
| SMBG Frequency | race (white/nonwhite), work status, admission service, reason for hospitalization, neuropathy |
| SMBG > 300 mg/dl | education, homeownership, neuropathy, cerebrovascular disease |

**Supplementary Table 3. Analysis of Availability of HbA1c at Follow-up by Baseline Characteristics**

| **Baseline Characteristic** | **HbA1c available 12 weeks** | | ***P*-value** | **HbA1c available 24 weeks** | | ***P*-value** |
| --- | --- | --- | --- | --- | --- | --- |
|  | **Yes** | **No** |  | **Yes** | **No** |  |
| DOS group | 54 (50) | 26 (55) | .60 | 44 (49) | 35 (54) | .63 |
| Age | 52 (44.3, 59.5) | 50 (43, 59.5) | .61 | 52 (45, 59) | 50 (42, 61) | .55 |
| Male | 44 (41) | 24 (49) | .39 | 38 (43) | 30 (45) | .87 |
| White race | 61 (56) | 23 (47) | .30 | 49 (55) | 34 (51) | .63 |
| Diabetes duration | 11 (7, 20) | 10 (7.3, 19.5) | .87 | 11 (6, 20) | 10.5 (8.8, 18.5) | .77 |
| Married | 47 (44) | 18 (37) | .49 | 34 (38) | 31 (46) | .33 |
| Employed | 41 (38) | 22 (45) | .48 | 31 (35) | 31 (46) | .19 |
| Insulin use prior to admission | 91 (84) | 36 (73) | .13 | 73 (82) | 53 (79) | .68 |
| Charlson comorbidity | 3 (2, 4.8) | 3 (1, 4.5) | .52 | 3 (2, 5) | 3 (1, 4) | .65 |
| Baseline HbA1c | 10.6 (9.4, 11.8) | 11.2 (10, 12.3) | .09 | 10.8 (9.5, 11.8) | 10.9 (9.7, 12.3) | .34 |

Data are reported as N (%) or median (25%-75%). DOS=Discharge Order Set

**Supplementary Table 4: Follow-up Data**

|  | **ESC** | **DOS** | **Diff/OR^a^** | ***P*-value^b^** | **ESC** | **DOS** | **Diff/OR^a^** | ***P*-value^b^** |
| --- | --- | --- | --- | --- | --- | --- | --- | --- |
|  | **2 weeks** | | | | **6 weeks** | | | |
| **Disposition** | 41 | 35 | - | - | 40 | 34 | - | - |
| **Follow-up** |  |  |  |  |  |  |  |  |
| Primary Care^c^ | 22 (46.8) | 29 (64.4) | - | .39 | 34 (77.3) | 29 (70.7) | - | >.99 |
| Endocrinology | 11 (24.4) | 8 (21.1) | 0.78 (0.14, 4.42) | >.99 | 14 (31.8) | 8 (21.1) | 0.43 (0.08, 2.48) | >.99 |
| **Emergency Visit^c^** | 13 (29.6) | 9 (24.3) | - | >.99 | 27 (61.4) | 21 (58.3) | - | >.99 |
| **Readmission** | 6 (13.6) | 6 (16.7) | 1.51 (0.20, 11.41) | >.99 | 14 (29.7) | 20 (44.4) | 2.26 (0.47, 10.87) | >.99 |
| **Total Daily Insulin** |  |  |  |  |  |  |  |  |
| Discharge (units) | 83.6 (55.5) | 82.8 (59.3) |  |  |  |  |  |  |
| Follow-up (units) | 84.7 (51.2) | 72.1 (51.5) |  |  | 95.4 (54.5) | 87.1 (68.2) |  |  |
| Change (units)^d^ | 0.6 (4.4) | -3.5 (4.8) | -5.8 (-19.7, 8.1) | >.99 | 5.8 (5.1) | 3.9 (5.2) | -2.5 (-18.4, 13.4) | >.99 |
| **Basal Insulin** |  |  |  |  |  |  |  |  |
| Discharge (units) | 55.2 (33.8) | 57.1 (43.1) |  |  |  |  |  |  |
| Follow-up (units) | 53.9 (31.8) | 52.1 (38.4) |  |  | 61.2 (38.1) | 54.5 (39.9) |  |  |
| Change (units)^d^ | -1.7 (3.2) | -0.1 (3.6) | -0.1 (-10.2,10.0) | >.99 | 3.2 (3.4) | -0.4 (3.6) | -0.4 (-12.0, 11.3) | >.99 |
| Increased^e^ | 6 (14.3) | 8 (25.0) | 2.06 (0.59, 7.19) | .51 | 9 (22.5) | 16 (48.5) | 3.53 (1.18, 10.62) | .07 |
| Decreased^c,e^ | 8 (19.1) | 6 (18.8) | - | >.99 | 6 (15.0) | 5 (15.2) | - | >.99 |
| Adherence^c.f^ | 38 (90.5) | 32 (97.0) | - | .84 | 35 (87.5) | 33 (97.1) | - | .84 |
| **Hypoglycemia** |  |  |  |  |  |  |  |  |
| <70 g/dl^c,g^ | 8 (19.5) | 2 (6.1) | - | .65 | 7 (18.0) | 2 (5.9) | - | .65 |
| <54 mg/ dl^c,g^ | 1 (2.4) | 0 (0) | - | >.99 | 2 (5.1) | 0 (0) | - | >.99 |
| Severe^c,g,h^ | 3 (7.7) | 1 (3.0) | - | >.99 | 2 (5.3) | 0 (0) | - | >.99 |
|  | **12 weeks** | | | | **24 weeks** | | | |
| **Disposition** | 38 | 45 | - | - | 30 | 32 | - | - |
| **Follow-up** |  |  |  |  |  |  |  |  |
| Primary Care^c^ | 50 (92.6) | 53 (82.8) | - | .50 | 55 (96.5) | 57 (95.0) | 1.00 | - |
| Endocrinology | 23 (48.9) | 28 (53.9) | 1.50 (0.35, 6.42) | >.99 | 28 (65.1) | 31 (60.8) | 0.80 (0.18, 3.61) | >.99 |
| **Emergency Visit^c^** | 44 (89.8) | 47 (82.5) | - | >.99 | 53 (100) | 53 (94.6) | - | .97 |
| **Readmission** | 24 (50.0) | 30 (49.2) | 1.03 (0.25, 4.28) | >.99 | 33 (67.4) | 36 (72.0) | 1.40 (0.30, 6.49) | >.99 |
| **Total Daily Insulin** |  |  |  |  |  |  |  |  |
| Discharge (units) |  |  |  |  |  |  |  |  |
| Follow-up (units) | 102.7 (52.1) | 95.3 (74.9) |  |  | 86.9 (50.2) | 94.6 (90.5) |  |  |
| Change (units)^d^ | 8.5 (5.4) | 11.5 (5.1) | 0.6 (-15.4, 16.6) | >.99 | 0.8 (8.2) | 16.9 (7.8) | 17.3 (-7.4, 42.0) | .67 |
| **Basal Insulin** |  |  |  |  |  |  |  |  |
| Follow-up (units) | 64.1 (33.5) | 64.6 (48.0) |  |  | 60.1 (32.1) | 65.8 (69.1) |  |  |
| Change (units)^d^ | 4.1 (3.9) | 6.4 (3.6) | 0.5 (-11.3, 12.2) | >.99 | 2.8 (6.9) | 11.2 (6.6) | 2.4 (-18.6,23.4) | >.99 |
| Increased^e^ | 8 (21.1) | 25 (53.2) | **4.70 (1.63, 13.52)** | **.02** | 11 (37.9) | 13 (40.6) | 1.09 (0.36 3.31) | .88 |
| Decreased^c,e^ | 1 (2.6) | 13 (27.7) | - | **.009** | 4 (13.8) | 7 (21.9) | - | >.99 |
| Adherence^c.f^ | 30 (79.0) | 42 (89.4) | - | .84 | 25 (83.3) | 25 (80.7) | - | >.99 |
| **Hypoglycemia** |  |  |  |  |  |  |  |  |
| <70 g/dl^c,g^ | 9 (23.7) | 15 (32.6) | - | .94 | 9 (30.0) | 8 (25.8) | - | .94 |
| <54 mg/ dl^c,g^ | 7 (18.4) | 3 (6.5) | - | .69 | 4 (13.3) | 2 (6.5) | - | >.99 |
| Severe^c,g,h^ | 1 (2.6) | 3 (7.0) | - | >.99 | 4 (13.3) | 1 (3.6) | - | >.99 |

Data reported as N (%), median (25-75%), or mean (SD). ^a^Difference (for continuous variables and SMBG frequency) or odds ratio (for binary variables) adjusted for confounders and the 95% confidence interval. The confounders used in each analysis are provided in Supplemental Table 2. ^b^Holm’s method used to correct p-values for tests at each time point. ^c^Regression model could not be fit due to small cell counts; Fisher’s exact test was performed. ^d^Average change from discharge (SE); estimated using a linear mixed model. ^e^Since previous visit. ^f^Defined as >80% of doses in previous week.  ^g^Hypoglycemia data were self-reported since previous visit. ^h^Events requiring external assistance. There was one death by 24 weeks in the DOS arm. Data for follow-up clinic visits, emergency department visits, and readmissions were collected at study visits and when possible, extracted from the electronic medical record. All other data were obtained from study visits only. ESC=enhanced standard care, DOS=discharge order set, SMBG=self-monitored blood glucose.

**Supplementary Table 5. Follow-up Non-insulin Medications**

|  | **12 weeks** | | | **24 weeks** | | |
| --- | --- | --- | --- | --- | --- | --- |
| **New Medication** | **ESC** | **DOS** | ***P*-value** | **ESC** | **DOS** | ***P*-value** |
| Metformin | 4 (10.5) | 5 (11.1) | >.99 | 5 (16.7) | 1 (3.1) | .10 |
| Sulfonylurea | 1 (2.6) | 0 | .46 | 3 (10.0) | 2 (6.3) | .67 |
| SGLT2-inhibitor | 1 (2.6) | 7 (15.6) | .06 | 1 (3.3) | 5 (15.6) | .20 |
| DPP-4 inhibitor | 1 (2.6) | 3 (6.7) | .62 | 0 | 1 (3.1) | >.99 |
| GLP-1 receptor agonist | 1 (2.6) | 1 (2.2) | >.99 | 4 (13.3) | 4 (12.5) | >.99 |
| Thiazolidinedione | 0 | 1 (2.2) | >.99 | 0 | 0 | - |
| Any non-insulin therapy | 7 (18) | 13 (29) | .31 | 9 (30.0) | 12 (37.5) | .59 |

Data reported as N (%). SGLT2=sodium-glucose cotransporter-2, DPP-4=dipeptidyl peptidase-4, GLP-1=glucagon-like peptide-1. ESC=enhanced standard care, DOS=discharge order set, SMBG=self-monitored blood glucose.
