# Supplementary material for: Effectiveness of a Diabetes-Focused Electronic Discharge Order Set and Postdischarge Nursing Support Among Poorly Controlled Hospitalized Patients: Randomized Controlled Trial
Source: JMIR Diabetes. 2022 Jul 26;7(3):e33401. doi: 10.2196/33401 (PMC9364166; doi:10.2196/33401)
Supplement: Multimedia Appendix 2 [file diabetes_v7i3e33401_app2.pptx]

## Slide 1
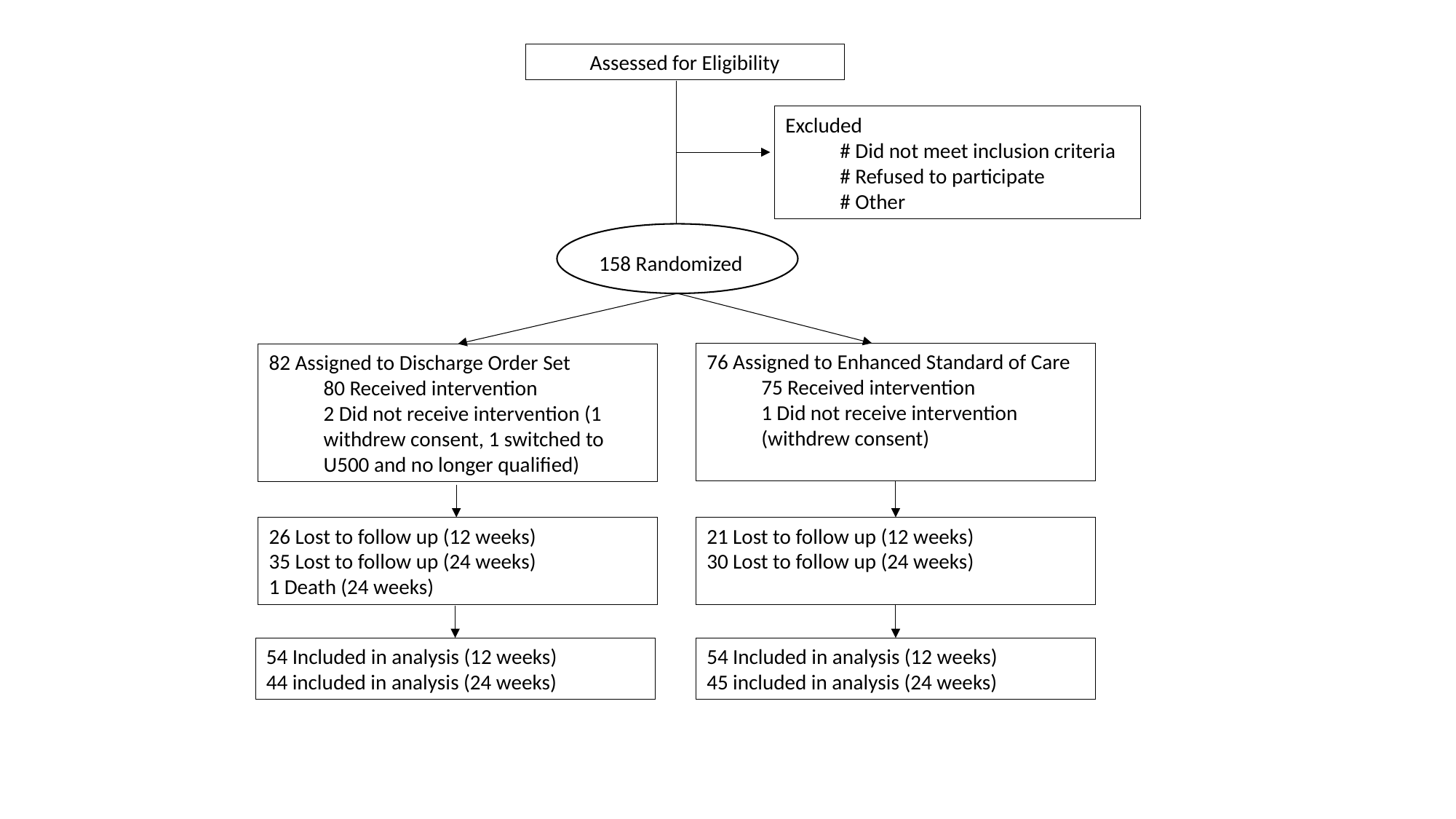

Assessed for Eligibility
Excluded
# Did not meet inclusion criteria
# Refused to participate
# Other
158 Randomized
76 Assigned to Enhanced Standard of Care
75 Received intervention
1 Did not receive intervention (withdrew consent)
82 Assigned to Discharge Order Set
80 Received intervention
2 Did not receive intervention (1 withdrew consent, 1 switched to U500 and no longer qualified)
26 Lost to follow up (12 weeks)
35 Lost to follow up (24 weeks)
1 Death (24 weeks)
21 Lost to follow up (12 weeks)
30 Lost to follow up (24 weeks)
54 Included in analysis (12 weeks)
44 included in analysis (24 weeks)
54 Included in analysis (12 weeks)
45 included in analysis (24 weeks)
