# Supplementary material for: Effectiveness of a Diabetes-Focused Electronic Discharge Order Set and Postdischarge Nursing Support Among Poorly Controlled Hospitalized Patients: Randomized Controlled Trial
Source: JMIR Diabetes. 2022 Jul 26;7(3):e33401. doi: 10.2196/33401 (PMC9364166; doi:10.2196/33401)
Supplement: Multimedia Appendix 3 [file diabetes_v7i3e33401_app3.pdf]

CONSORT-EHEALTH (V 1.6.1) - Submission/Publication Form

The CONSORT-EHEALTH checklist is intended for authors of randomized trials evaluating web-based and Internet-based applications/interventions, including mobile interventions, electronic games (incl multiplayer games), social media, certain telehealth applications, and other interactive and/or networked electronic applications. Some of the items (e.g. all subitems under item 5 - description of the intervention) may also be applicable for other study designs.

The goal of the CONSORT EHEALTH checklist and guideline is to be
a) a guide for reporting for authors of RCTs,
b) to form a basis for appraisal of an ehealth trial (in terms of validity)

CONSORT-EHEALTH items/subitems are MANDATORY reporting items for studies published in the Journal of Medical Internet Research and other journals / scientific societies endorsing the checklist.

Items numbered 1., 2., 3., 4a., 4b etc are original CONSORT or CONSORT-NPT (non-pharmacologic treatment) items.
Items with Roman numerals (i., ii, iii, iv etc.) are CONSORT-EHEALTH extensions/clarifications.

As the CONSORT-EHEALTH checklist is still considered in a formative stage, we would ask that you also RATE ON A SCALE OF 1-5 how important/useful you feel each item is FOR THE PURPOSE OF THE CHECKLIST and reporting guideline (optional).

Mandatory reporting items are marked with a red *.
In the textboxes, either copy & paste the relevant sections from your manuscript into this form - please include any quotes from your manuscript in QUOTATION MARKS,
or answer directly by providing additional information not in the manuscript, or elaborating on why the item was not relevant for this study.

YOUR ANSWERS WILL BE PUBLISHED AS A SUPPLEMENTARY FILE TO YOUR PUBLICATION

You're editing your response. Sharing this URL allows others to also edit your response. FILL OUT A NEW RESPONSE

proper spelling and grammar, use correct capitalization, and avoid abbreviations.

DO NOT FORGET TO SAVE AS PDF _AND_ CLICK THE SUBMIT BUTTON SO YOUR ANSWERS ARE IN OUR DATABASE !!!

Citation Suggestion (if you append the pdf as Appendix we suggest to cite this paper in the caption):
Eysenbach G, CONSORT-EHEALTH Group
CONSORT-EHEALTH: Improving and Standardizing Evaluation Reports of Web-based and Mobile Health Interventions
J Med Internet Res 2011;13(4):e126
URL: http://www.jmir.org/2011/4/e126/
doi: 10.2196/jmir.1923
PMID: 22209829

kmdungan@gmail.com (not shared) Switch account

Resubmit to save

* Required

Your name *

First Last

Kathleen Dungan

Primary Affiliation (short), City, Country *

University of Toronto, Toronto, Canada

The Ohio State University, Columbus, USA

You're editing your response. Sharing this URL allows others to also edit your response.

FILL OUT A NEW RESPONSE

Your e-mail address *

abc@gmail.com

kathleen.dungan@osumc.edu

Title of your manuscript *

Provide the (draft) title of your manuscript.

Effectiveness of a diabetes focused electronic discharge orderset and post-discharge nursing support among poorly controlled hospitalized patients: A randomized controlled trial

Name of your App/Software/Intervention *

If there is a short and a long/alternate name, write the short name first and add the long name in brackets.

Diabetes discharge order set

Evaluated Version (if any)

e.g. "V1", "Release 2017-03-01", "Version 2.0.27913"

Not applicable

You're editing your response. Sharing this URL allows others to also edit your response. FILL OUT A NEW RESPONSE

Language(s) *

What language is the intervention/app in? If multiple languages are available, separate by comma (e.g. "English, French")

English

URL of your Intervention Website or App

e.g. a direct link to the mobile app on app in appstore (itunes, Google Play), or URL of the website. If the intervention is a DVD or hardware, you can also link to an Amazon page.

https://www.epic.com/

URL of an image/screenshot (optional)

Your answer

You're editing your response. Sharing this URL allows others to also edit your response.

FILL OUT A NEW RESPONSE

Accessibility *

Can an enduser access the intervention presently?

- ○ access is free and open
- ◉ access only for special usergroups, not open
- ○ access is open to everyone, but requires payment/subscription/in-app purchases
- ○ app/intervention no longer accessible
- ○ Other:

Primary Medical Indication/Disease/Condition *

e.g. "Stress", "Diabetes", or define the target group in brackets after the condition, e.g. "Autism (Parents of children with)", "Alzheimers (Informal Caregivers of)"

type 2 diabetes (hospitalized)

Primary Outcomes measured in trial *

comma-separated list of primary outcomes reported in the trial

HbA1c at 24 weeks

You're editing your response. Sharing this URL allows others to also edit your response. FILL OUT A NEW RESPONSE

Secondary/other outcomes

Are there any other outcomes the intervention is expected to affect?

Completeness and accuracy of discharge prescriptions related to diabetes, fasting glucose at 12, 24 weeks, HbA1c at 12 weeks, individualized A1c target at 12, 24 weeks.

Recommended "Dose" *

What do the instructions for users say on how often the app should be used?

- ○ Approximately Daily
- ○ Approximately Weekly
- ○ Approximately Monthly
- ○ Approximately Yearly
- ○ "as needed"
- ◉ Other: hospital discharge

You're editing your response. Sharing this URL allows others to also edit your response. FILL OUT A NEW RESPONSE

Approx. Percentage of Users (starters) still using the app as recommended after 3 months *

- (selected) unknown / not evaluated
- 0-10%
- 11-20%
- 21-30%
- 31-40%
- 41-50%
- 51-60%
- 61-70%
- 71%-80%
- 81-90%
- 91-100%
- Other:

You're editing your response. Sharing this URL allows others to also edit your response.

FILL OUT A NEW RESPONSE

Overall, was the app/intervention effective? *

- ○ yes: all primary outcomes were significantly better in intervention group vs control
- ○ partly: SOME primary outcomes were significantly better in intervention group vs control
- ○ no statistically significant difference between control and intervention
- ○ potentially harmful: control was significantly better than intervention in one or more outcomes
- ○ inconclusive: more research is needed
- ◉ Other: The discharge order set did not improve the primary outcome (HbA1c

Article Preparation Status/Stage *

At which stage in your article preparation are you currently (at the time you fill in this form)

- ○ not submitted yet - in early draft status
- ○ not submitted yet - in late draft status, just before submission
- ○ submitted to a journal but not reviewed yet
- ◉ submitted to a journal and after receiving initial reviewer comments
- ○ submitted to a journal and accepted, but not published yet
- ○ published
- ○ Other:

You're editing your response. Sharing this URL allows others to also edit your response. FILL OUT A NEW RESPONSE

Journal *

If you already know where you will submit this paper (or if it is already submitted), please provide the journal name (if it is not JMIR, provide the journal name under "other")

- ( ) not submitted yet / unclear where I will submit this
- ( ) Journal of Medical Internet Research (JMIR)
- ( ) JMIR mHealth and UHealth
- ( ) JMIR Serious Games
- ( ) JMIR Mental Health
- ( ) JMIR Public Health
- ( ) JMIR Formative Research
- (●) Other JMIR sister journal
- ( ) Other:

Is this a full powered effectiveness trial or a pilot/feasibility trial? *

- ( ) Pilot/feasibility
- (●) Fully powered

You're editing your response. Sharing this URL allows others to also edit your response. FILL OUT A NEW RESPONSE

Manuscript tracking number *

If this is a JMIR submission, please provide the manuscript tracking number under "other" (The ms tracking number can be found in the submission acknowledgement email, or when you login as author in JMIR. If the paper is already published in JMIR, then the ms tracking number is the four-digit number at the end of the DOI, to be found at the bottom of each published article in JMIR)

- [ ] no ms number (yet) / not (yet) submitted to / published in JMIR
- [x] Other: 33401

TITLE AND ABSTRACT

1a) TITLE: Identification as a randomized trial in the title

1a) Does your paper address CONSORT item 1a? *

I.e does the title contain the phrase "Randomized Controlled Trial"? (if not, explain the reason under "other")

- [x] yes
- [ ] Other:

You're editing your response. Sharing this URL allows others to also edit your response. FILL OUT A NEW RESPONSE

1a-i) Identify the mode of delivery in the title

Identify the mode of delivery. Preferably use “web-based” and/or “mobile” and/or “electronic game” in the title. Avoid ambiguous terms like “online”, “virtual”, “interactive”. Use “Internet-based” only if Intervention includes non-web-based Internet components (e.g. email), use “computer-based” or “electronic” only if offline products are used. Use “virtual” only in the context of “virtual reality” (3-D worlds). Use “online” only in the context of “online support groups”. Complement or substitute product names with broader terms for the class of products (such as “mobile” or “smart phone” instead of “iphone”), especially if the application runs on different platforms.

	1	2	3	4	5	
subitem not at all important	○	○	○	○	◉	essential

Clear selection

Does your paper address subitem 1a-i? *

Copy and paste relevant sections from manuscript title (include quotes in quotation marks "like this" to indicate direct quotes from your manuscript), or elaborate on this item by providing additional information not in the ms, or briefly explain why the item is not applicable/relevant for your study

Title contains "electronic discharge orderset"

1a-ii) Non-web-based components or important co-interventions in title

Mention non-web-based components or important co-interventions in title, if any (e.g., “with telephone support”).

	1	2	3	4	5	
subitem not at all important	○	○	○	○	◉	essential

You're editing your response. Sharing this URL allows others to also edit your response.

FILL OUT A NEW RESPONSE

Does your paper address subitem 1a-ii?

Copy and paste relevant sections from manuscript title (include quotes in quotation marks "like this" to indicate direct quotes from your manuscript), or elaborate on this item by providing additional information not in the ms, or briefly explain why the item is not applicable/relevant for your study

Title contains "post-discharge nursing support"

1a-iii) Primary condition or target group in the title

Mention primary condition or target group in the title, if any (e.g., “for children with Type I Diabetes”) Example: A Web-based and Mobile Intervention with Telephone Support for Children with Type I Diabetes: Randomized Controlled Trial

	1	2	3	4	5	
subitem not at all important	○	○	○	○	○	essential

Does your paper address subitem 1a-iii? *

Copy and paste relevant sections from manuscript title (include quotes in quotation marks "like this" to indicate direct quotes from your manuscript), or elaborate on this item by providing additional information not in the ms, or briefly explain why the item is not applicable/relevant for your study

Title contains "diabetes" and "poorly controlled hospitalized patients"

1b) ABSTRACT: Structured summary of trial design, methods, results, and conclusions

NPT extension: Description of experimental treatment, comparator, care providers, centers, and blinding [illegible]

You're editing your response. Sharing this URL allows others to also edit your response.

FILL OUT A NEW RESPONSE

1b-i) Key features/functionalities/components of the intervention and comparator in the METHODS section of the ABSTRACT

Mention key features/functionalities/components of the intervention and comparator in the abstract. If possible, also mention theories and principles used for designing the site. Keep in mind the needs of systematic reviewers and indexers by including important synonyms. (Note: Only report in the abstract what the main paper is reporting. If this information is missing from the main body of text, consider adding it)

	1	2	3	4	5	
subitem not at all important	○	○	○	○	◉	essential

Clear selection

Does your paper address subitem 1b-i? *

Copy and paste relevant sections from the manuscript abstract (include quotes in quotation marks "like this" to indicate direct quotes from your manuscript), or elaborate on this item by providing additional information not in the ms, or briefly explain why the item is not applicable/relevant for your study

"This is a randomized single center study comparing an electronic DOS and nurse phone calls to enhanced standard care (ESC) in hospitalized insulin-requiring patients with T2D."

You're editing your response. Sharing this URL allows others to also edit your response. FILL OUT A NEW RESPONSE

1b-ii) Level of human involvement in the METHODS section of the ABSTRACT

Clarify the level of human involvement in the abstract, e.g., use phrases like “fully automated” vs. “therapist/nurse/care provider/physician-assisted” (mention number and expertise of providers involved, if any). (Note: Only report in the abstract what the main paper is reporting. If this information is missing from the main body of text, consider adding it)

	1	2	3	4	5	
subitem not at all important	○	○	◉	○	○	essential

Clear selection

Does your paper address subitem 1b-ii?

Copy and paste relevant sections from the manuscript abstract (include quotes in quotation marks "like this" to indicate direct quotes from your manuscript), or elaborate on this item by providing additional information not in the ms, or briefly explain why the item is not applicable/relevant for your study

"an electronic discharge order set (DOS) and nurse phone calls".

You're editing your response. Sharing this URL allows others to also edit your response.

FILL OUT A NEW RESPONSE

1b-iii) Open vs. closed, web-based (self-assessment) vs. face-to-face assessments in the METHODS section of the ABSTRACT

Mention how participants were recruited (online vs. offline), e.g., from an open access website or from a clinic or a closed online user group (closed usergroup trial), and clarify if this was a purely web-based trial, or there were face-to-face components (as part of the intervention or for assessment). Clearly say if outcomes were self-assessed through questionnaires (as common in web-based trials). Note: In traditional offline trials, an open trial (open-label trial) is a type of clinical trial in which both the researchers and participants know which treatment is being administered. To avoid confusion, use “blinded” or “unblinded” to indicated the level of blinding instead of “open”, as “open” in web-based trials usually refers to “open access” (i.e. participants can self-enrol). (Note: Only report in the abstract what the main paper is reporting. If this information is missing from the main body of text, consider adding it)

	1	2	3	4	5	
subitem not at all important	○	○	◉	○	○	essential

Clear selection

Does your paper address subitem 1b-iii?

Copy and paste relevant sections from the manuscript abstract (include quotes in quotation marks "like this" to indicate direct quotes from your manuscript), or elaborate on this item by providing additional information not in the ms, or briefly explain why the item is not applicable/relevant for your study

"open label single center study " performed only among patients hospitalized at the site."

You're editing your response. Sharing this URL allows others to also edit your response.

FILL OUT A NEW RESPONSE

1b-iv) RESULTS section in abstract must contain use data

Report number of participants enrolled/assessed in each group, the use/uptake of the intervention (e.g., attrition/adherence metrics, use over time, number of logins etc.), in addition to primary/secondary outcomes. (Note: Only report in the abstract what the main paper is reporting. If this information is missing from the main body of text, consider adding it)

	1	2	3	4	5	
subitem not at all important	○	○	○	○	◉	essential

Clear selection

Does your paper address subitem 1b-iv?

Copy and paste relevant sections from the manuscript abstract (include quotes in quotation marks "like this" to indicate direct quotes from your manuscript), or elaborate on this item by providing additional information not in the ms, or briefly explain why the item is not applicable/relevant for your study

"a total of 158 subjects were enrolled (DOS=82, ESC=76), 155 of whom had discharge data."

1b-v) CONCLUSIONS/DISCUSSION in abstract for negative trials

Conclusions/Discussions in abstract for negative trials: Discuss the primary outcome - if the trial is negative (primary outcome not changed), and the intervention was not used, discuss whether negative results are attributable to lack of uptake and discuss reasons. (Note: Only report in the abstract what the main paper is reporting. If this information is missing from the main body of text, consider adding it)

	1	2	3	4	5	
subitem not at all important	○	○	○	○	◉	essential

You're editing your response. Sharing this URL allows others to also edit your response.

FILL OUT A NEW RESPONSE

Does your paper address subitem 1b-v?

Copy and paste relevant sections from the manuscript abstract (include quotes in quotation marks "like this" to indicate direct quotes from your manuscript), or elaborate on this item by providing additional information not in the ms, or briefly explain why the item is not applicable/relevant for your study

"An intervention that included a DOS plus post-discharge nurse phone calls resulted in more complete discharge prescriptions. Assessment of post-discharge outcomes was limited due to loss of long-term follow-up but suggests possible benefit in glucose control. "

INTRODUCTION

2a) In INTRODUCTION: Scientific background and explanation of rationale

2a-i) Problem and the type of system/solution

Describe the problem and the type of system/solution that is object of the study: intended as stand-alone intervention vs. incorporated in broader health care program? Intended for a particular patient population? Goals of the intervention, e.g., being more cost-effective to other interventions, replace or complement other solutions? (Note: Details about the intervention are provided in "Methods" under 5)

	1	2	3	4	5	
subitem not at all important	○	○	○	○	◉	essential

Clear selection

You're editing your response. Sharing this URL allows others to also edit your response. FILL OUT A NEW RESPONSE

Does your paper address subitem 2a-i? *

Copy and paste relevant sections from the manuscript (include quotes in quotation marks "like this" to indicate direct quotes from your manuscript), or elaborate on this item by providing additional information not in the ms, or briefly explain why the item is not applicable/relevant for your study

"Despite being the most frequently utilized task specific order set during hospitalization [17], order sets have not been utilized to guide insulin use at hospital discharge [18]. In fact, preliminary studies at our institution demonstrated that a switch to a new electronic medical record platform resulted in an increase in unclear prescriptions for insulin at the time of discharge, in part due to the use of a free text field in insulin prescriptions [19]. "

2a-ii) Scientific background, rationale: What is known about the (type of) system

Scientific background, rationale: What is known about the (type of) system that is the object of the study (be sure to discuss the use of similar systems for other conditions/diagnoses, if appropiate), motivation for the study, i.e. what are the reasons for and what is the context for this specific study, from which stakeholder viewpoint is the study performed, potential impact of findings [2]. Briefly justify the choice of the comparator.

	1	2	3	4	5	
subitem not at all important	○	○	○	○	◉	essential

Clear selection

You're editing your response. Sharing this URL allows others to also edit your response. FILL OUT A NEW RESPONSE

Does your paper address subitem 2a-ii? *

Copy and paste relevant sections from the manuscript (include quotes in quotation marks "like this" to indicate direct quotes from your manuscript), or elaborate on this item by providing additional information not in the ms, or briefly explain why the item is not applicable/relevant for your study

"In a Society of Hospital Medicine Survey, only one fourth of hospitals were supported with written protocols to standardize medication, education, equipment, and follow-up instructions for hospitalized patients with diabetes [16]."

2b) In INTRODUCTION: Specific objectives or hypotheses

Does your paper address CONSORT subitem 2b? *

Copy and paste relevant sections from the manuscript (include quotes in quotation marks "like this" to indicate direct quotes from your manuscript), or elaborate on this item by providing additional information not in the ms, or briefly explain why the item is not applicable/relevant for your study

"This study will assess whether a diabetes focused inpatient discharge order set (DOS) with nurse follow-up calls can improve post-discharge outcomes compared to enhanced standard care (ESC) among hospitalized patients with insulin-requiring type 2 diabetes."

METHODS

3a) Description of trial design (such as parallel, factorial) including allocation ratio

You're editing your response. Sharing this URL allows others to also edit your response. FILL OUT A NEW RESPONSE

Does your paper address CONSORT subitem 3a? *

Copy and paste relevant sections from the manuscript (include quotes in quotation marks "like this" to indicate direct quotes from your manuscript), or elaborate on this item by providing additional information not in the ms, or briefly explain why the item is not applicable/relevant for your study

"This is a single center 24 week randomized open label parallel group controlled trial."
"Randomization to the DOS or ESC was performed 1:1... "

3b) Important changes to methods after trial commencement (such as eligibility criteria), with reasons

Does your paper address CONSORT subitem 3b? *

Copy and paste relevant sections from the manuscript (include quotes in quotation marks "like this" to indicate direct quotes from your manuscript), or elaborate on this item by providing additional information not in the ms, or briefly explain why the item is not applicable/relevant for your study

"However, study enrollment halted in March 2020 with the COVID-19 crisis and concerns with the feasibility of continuing to enroll and conduct study visits. "

You're editing your response. Sharing this URL allows others to also edit your response. FILL OUT A NEW RESPONSE

3b-i) Bug fixes, Downtimes, Content Changes

Bug fixes, Downtimes, Content Changes: ehealth systems are often dynamic systems. A description of changes to methods therefore also includes important changes made on the intervention or comparator during the trial (e.g., major bug fixes or changes in the functionality or content) (5-iii) and other “unexpected events” that may have influenced study design such as staff changes, system failures/downtimes, etc. [2].

	1	2	3	4	5	
subitem not at all important	○	○	●	○	○	essential

Clear selection

Does your paper address subitem 3b-i?

Copy and paste relevant sections from the manuscript (include quotes in quotation marks "like this" to indicate direct quotes from your manuscript), or elaborate on this item by providing additional information not in the ms, or briefly explain why the item is not applicable/relevant for your study

No-there were no content changes during the trial.

4a) Eligibility criteria for participants

You're editing your response. Sharing this URL allows others to also edit your response.

FILL OUT A NEW RESPONSE

Does your paper address CONSORT subitem 4a? *

Copy and paste relevant sections from the manuscript (include quotes in quotation marks "like this" to indicate direct quotes from your manuscript), or elaborate on this item by providing additional information not in the ms, or briefly explain why the item is not applicable/relevant for your study

"Inclusion criteria were hospitalized patients age 25-75 years with type 2 diabetes for at least 3 months duration, HA1c >8.5% (69 mmol/mol) within 3 months prior to enrollment, requiring at least 10 units of basal insulin per day while in the hospital, and able to provide informed consent. Age 25 was chosen to minimize the possibility of inadvertently including type 1 diabetes [20, 21] while age 75 was chosen in order to minimize the inclusion of patients who were less likely to discharge home [22]. Participants were required to have access to a phone or electronic messaging post-discharge. Exclusion criteria included inmates, pregnancy, inability to consent, or expected skilled nursing facility stay greater than 2 weeks duration."

4a-i) Computer / Internet literacy

Computer / Internet literacy is often an implicit “de facto” eligibility criterion - this should be explicitly clarified.

	1	2	3	4	5	
subitem not at all important	○	○	●	○	○	essential

Clear selection

You're editing your response. Sharing this URL allows others to also edit your response. FILL OUT A NEW RESPONSE

Does your paper address subitem 4a-i?

Copy and paste relevant sections from the manuscript (include quotes in quotation marks "like this" to indicate direct quotes from your manuscript), or elaborate on this item by providing additional information not in the ms, or briefly explain why the item is not applicable/relevant for your study

The DOS was directed at physicians and advanced practice providers and computer literacy was not a determinant of patient enrollment. However, patients were required to have some means of contact by the study nurse: "Participants were required to have access to a phone or electronic messaging post-discharge."

4a-ii) Open vs. closed, web-based vs. face-to-face assessments:

Open vs. closed, web-based vs. face-to-face assessments: Mention how participants were recruited (online vs. offline), e.g., from an open access website or from a clinic, and clarify if this was a purely web-based trial, or there were face-to-face components (as part of the intervention or for assessment), i.e., to what degree got the study team to know the participant. In online-only trials, clarify if participants were quasi-anonymous and whether having multiple identities was possible or whether technical or logistical measures (e.g., cookies, email confirmation, phone calls) were used to detect/prevent these.

	1	2	3	4	5	
subitem not at all important	○	○	○	○	◉	essential

Clear selection

You're editing your response. Sharing this URL allows others to also edit your response. FILL OUT A NEW RESPONSE

Does your paper address subitem 4a-ii? *

Copy and paste relevant sections from the manuscript (include quotes in quotation marks "like this" to indicate direct quotes from your manuscript), or elaborate on this item by providing additional information not in the ms, or briefly explain why the item is not applicable/relevant for your study

"Participants were identified through daily screening of the inpatient medical and surgical services throughout the institution and were enrolled between 5/1/2018-3/4/2020. Permission was obtained from the attending physician of the inpatient service prior to approaching the patient in person for consent."
"All participants in both treatment groups received a phone call at 2 weeks and 6 weeks following discharge, in which data related to ambulatory and inpatient encounters, glucose monitoring and insulin use were collected. Basal insulin adherence was defined as >80% of doses taken in the previous week. The participants in the DOS received follow-up telephone calls by the study nurse to facilitate ongoing basal insulin dose titration and hospital follow-up. The nurse had a basic understanding of diabetes but was not a Certified Diabetes Care and Education Specialist. In the ESC group, follow-up telephone calls and visits were conducted on the same schedule as the ESC group but were conducted by the study coordinator for the purposes of information gathering only. In-person visits at 12 weeks and 24 weeks were conducted by the coordinator in the ESC group and by the nurse with or without the coordinator in the DOS group. "

4a-iii) Information giving during recruitment

Information given during recruitment. Specify how participants were briefed for recruitment and in the informed consent procedures (e.g., publish the informed consent documentation as appendix, see also item X26), as this information may have an effect on user self-selection, user expectation and may also bias results.

	1	2	3	4	5	
subitem not at all important	○	○	○	○	◉	essential

Clear selection

You're editing your response. Sharing this URL allows others to also edit your response. FILL OUT A NEW RESPONSE

Does your paper address subitem 4a-iii?

Copy and paste relevant sections from the manuscript (include quotes in quotation marks "like this" to indicate direct quotes from your manuscript), or elaborate on this item by providing additional information not in the ms, or briefly explain why the item is not applicable/relevant for your study

"Participants were identified through daily screening of the inpatient medical and surgical services throughout the institution and were enrolled between 5/1/2018-3/4/2020. Permission was obtained from the attending physician of the inpatient service prior to approaching the patient in person for consent. "

4b) Settings and locations where the data were collected

Does your paper address CONSORT subitem 4b? *

Copy and paste relevant sections from the manuscript (include quotes in quotation marks "like this" to indicate direct quotes from your manuscript), or elaborate on this item by providing additional information not in the ms, or briefly explain why the item is not applicable/relevant for your study

"This is a single center 24 week randomized open label parallel group controlled trial. Inclusion criteria were hospitalized patients..."
"All participants in both treatment groups received a phone call at 2 weeks and 6 weeks following discharge."
"In-person visits at 12 weeks and 24 weeks..."

You're editing your response. Sharing this URL allows others to also edit your response.

FILL OUT A NEW RESPONSE

4b-i) Report if outcomes were (self-)assessed through online questionnaires

Clearly report if outcomes were (self-)assessed through online questionnaires (as common in web-based trials) or otherwise.

	1	2	3	4	5	
subitem not at all important	○	○	◉	○	○	essential

Clear selection

Does your paper address subitem 4b-i? *

Copy and paste relevant sections from the manuscript (include quotes in quotation marks "like this" to indicate direct quotes from your manuscript), or elaborate on this item by providing additional information not in the ms, or briefly explain why the item is not applicable/relevant for your study

Most outcomes were not self-assessed.
"The primary outcome was the change in HbA1c from baseline to 24 weeks post-discharge."
"Secondary outcomes related to hospital discharge included proportion of patients with new prescriptions for insulin and related supplies and clear discharge patient orders instructions, including correct frequency, no jargon or technical terms, correct quantity dispensed and refills using the following definitions..."
"Post-discharge, self-reported insulin dosing and hypoglycemia were solicited. Secondary outcomes included HbA1c at 12 weeks, fasting glucose at 12 and 24 weeks, and proportion of subjects achieving HbA1c <7% (53 mmol/mol), < 6.5% (48 mmol/mol), or individualized HbA1c target, defined using Healthcare Effectiveness Data and Information Set (HEDIS) criteria.[21] "

You're editing your response. Sharing this URL allows others to also edit your response.

FILL OUT A NEW RESPONSE

4b-ii) Report how institutional affiliations are displayed

Report how institutional affiliations are displayed to potential participants [on ehealth media], as affiliations with prestigious hospitals or universities may affect volunteer rates, use, and reactions with regards to an intervention.(Not a required item – describe only if this may bias results)

	1	2	3	4	5	
subitem not at all important	○	○	●	○	○	essential

Clear selection

Does your paper address subitem 4b-ii?

Copy and paste relevant sections from the manuscript (include quotes in quotation marks "like this" to indicate direct quotes from your manuscript), or elaborate on this item by providing additional information not in the ms, or briefly explain why the item is not applicable/relevant for your study

This is not applicable for patient enrollment as the DOS was directed at clinicians.

5) The interventions for each group with sufficient details to allow replication, including how and when they were actually administered

You're editing your response. Sharing this URL allows others to also edit your response. FILL OUT A NEW RESPONSE

5-i) Mention names, credential, affiliations of the developers, sponsors, and owners

Mention names, credential, affiliations of the developers, sponsors, and owners [6] (if authors/evaluators are owners or developer of the software, this needs to be declared in a "Conflict of interest" section or mentioned elsewhere in the manuscript).

	1	2	3	4	5	
subitem not at all important	○	○	○	◉	○	essential

Clear selection

Does your paper address subitem 5-i?

Copy and paste relevant sections from the manuscript (include quotes in quotation marks "like this" to indicate direct quotes from your manuscript), or elaborate on this item by providing additional information not in the ms, or briefly explain why the item is not applicable/relevant for your study

"The DOS is embedded within the discharge navigator of the electronic medical record (EMR, Epic®)"

5-ii) Describe the history/development process

Describe the history/development process of the application and previous formative evaluations (e.g., focus groups, usability testing), as these will have an impact on adoption/use rates and help with interpreting results.

	1	2	3	4	5	
subitem not at all important	○	○	◉	○	○	essential

You're editing your response. Sharing this URL allows others to also edit your response.

FILL OUT A NEW RESPONSE

Does your paper address subitem 5-ii?

Copy and paste relevant sections from the manuscript (include quotes in quotation marks "like this" to indicate direct quotes from your manuscript), or elaborate on this item by providing additional information not in the ms, or briefly explain why the item is not applicable/relevant for your study

"The DOS was developed with the consultation of a multi-disciplinary team that included feeback from hospital medicine and diabetes specialists. "

5-iii) Revisions and updating

Revisions and updating. Clearly mention the date and/or version number of the application/intervention (and comparator, if applicable) evaluated, or describe whether the intervention underwent major changes during the evaluation process, or whether the development and/or content was “frozen” during the trial. Describe dynamic components such as news feeds or changing content which may have an impact on the replicability of the intervention (for unexpected events see item 3b).

	1	2	3	4	5	
subitem not at all important	○	○	○	○	◉	essential

Clear selection

Does your paper address subitem 5-iii?

Copy and paste relevant sections from the manuscript (include quotes in quotation marks "like this" to indicate direct quotes from your manuscript), or elaborate on this item by providing additional information not in the ms, or briefly explain why the item is not applicable/relevant for your study

"No modifications were made to the DOS during the study."

You're editing your response. Sharing this URL allows others to also edit your response. FILL OUT A NEW RESPONSE

5-iv) Quality assurance methods

Provide information on quality assurance methods to ensure accuracy and quality of information provided [1], if applicable.

	1	2	3	4	5	
subitem not at all important	○	○	◉	○	○	essential

Clear selection

Does your paper address subitem 5-iv?

Copy and paste relevant sections from the manuscript (include quotes in quotation marks "like this" to indicate direct quotes from your manuscript), or elaborate on this item by providing additional information not in the ms, or briefly explain why the item is not applicable/relevant for your study

As it was not clear whether prescribing teams were familiar with the use of glargine U300, basic guidance was provided in the enrollment note: "A basic description of Gla-300 was provided to hospital teams using a standard template, recommending that Gla-300 be provided in 1 or 2 injections per day at the same time of day (only pens with 1 unit dosing increments were available). A 1:1 initial dosing conversion from in-hospital administration of glargine U100 or detemir to outpatient Gla-300 was recommended."
"A discharge summary is sent to the primary care provider of record per routine practice. All patients were instructed to maintain a standardized study diary that records glucose levels and insulin dose by time of day as well as any hypoglycemic events and associated symptoms. "

You're editing your response. Sharing this URL allows others to also edit your response.

FILL OUT A NEW RESPONSE

5-v) Ensure replicability by publishing the source code, and/or providing screenshots/screen-capture video, and/or providing flowcharts of the algorithms used

Ensure replicability by publishing the source code, and/or providing screenshots/screen-capture video, and/or providing flowcharts of the algorithms used. Replicability (i.e., other researchers should in principle be able to replicate the study) is a hallmark of scientific reporting.

	1	2	3	4	5	
subitem not at all important	○	○	○	◉	○	essential

Clear selection

Does your paper address subitem 5-v?

Copy and paste relevant sections from the manuscript (include quotes in quotation marks "like this" to indicate direct quotes from your manuscript), or elaborate on this item by providing additional information not in the ms, or briefly explain why the item is not applicable/relevant for your study

"In the DOS, the following orders are accompanied by pre-selected options with additional cascading options to enhance decision support (Supplemental Table 1):..."
"The DOS is embedded within the discharge navigator of the electronic medical record (EMR, Epic®)."

You're editing your response. Sharing this URL allows others to also edit your response. FILL OUT A NEW RESPONSE

5-vi) Digital preservation

Digital preservation: Provide the URL of the application, but as the intervention is likely to change or disappear over the course of the years; also make sure the intervention is archived (Internet Archive, webcitation.org, and/or publishing the source code or screenshots/videos alongside the article). As pages behind login screens cannot be archived, consider creating demo pages which are accessible without login.

	1	2	3	4	5	
subitem not at all important	○	○	◉	○	○	essential

Clear selection

Does your paper address subitem 5-vi?

Copy and paste relevant sections from the manuscript (include quotes in quotation marks "like this" to indicate direct quotes from your manuscript), or elaborate on this item by providing additional information not in the ms, or briefly explain why the item is not applicable/relevant for your study

Unfortunately, Epic does not allow users to publish screen shots of work. However, we have submitted a mock-up of relevant content. "In the DOS, the following orders are accompanied by pre-selected options with additional cascading options to enhance decision support (Supplemental Table 1):"

You're editing your response. Sharing this URL allows others to also edit your response.

FILL OUT A NEW RESPONSE

5-vii) Access

Access: Describe how participants accessed the application, in what setting/context, if they had to pay (or were paid) or not, whether they had to be a member of specific group. If known, describe how participants obtained “access to the platform and Internet” [1]. To ensure access for editors/reviewers/readers, consider to provide a “backdoor” login account or demo mode for reviewers/readers to explore the application (also important for archiving purposes, see vi).

	1	2	3	4	5	
subitem not at all important	○	○	○	○	◉	essential

Clear selection

Does your paper address subitem 5-vii? *

Copy and paste relevant sections from the manuscript (include quotes in quotation marks "like this" to indicate direct quotes from your manuscript), or elaborate on this item by providing additional information not in the ms, or briefly explain why the item is not applicable/relevant for your study

"In the DOS arm, the primary team was instructed to verify and complete the discharge order set, which was launched by the study team."

You're editing your response. Sharing this URL allows others to also edit your response. FILL OUT A NEW RESPONSE

5-viii) Mode of delivery, features/functionalities/components of the intervention and comparator, and the theoretical framework

Describe mode of delivery, features/functionalities/components of the intervention and comparator, and the theoretical framework [6] used to design them (instructional strategy [1], behaviour change techniques, persuasive features, etc., see e.g., [7, 8] for terminology). This includes an in-depth description of the content (including where it is coming from and who developed it) [1],” whether [and how] it is tailored to individual circumstances and allows users to track their progress and receive feedback” [6]. This also includes a description of communication delivery channels and – if computer-mediated communication is a component – whether communication was synchronous or asynchronous [6]. It also includes information on presentation strategies [1], including page design principles, average amount of text on pages, presence of hyperlinks to other resources, etc. [1].

	1	2	3	4	5	
subitem not at all important	○	○	○	○	◉	essential

Clear selection

You're editing your response. Sharing this URL allows others to also edit your response. FILL OUT A NEW RESPONSE

Does your paper address subitem 5-viii? *

Copy and paste relevant sections from the manuscript (include quotes in quotation marks "like this" to indicate direct quotes from your manuscript), or elaborate on this item by providing additional information not in the ms, or briefly explain why the item is not applicable/relevant for your study

"The DOS is embedded within the discharge navigator of the electronic medical record (EMR, Epic®)."
"In the DOS, the following orders are accompanied by pre-selected options with additional cascading options to enhance decision support (Supplemental Table 1): ..."
"The discharge order set also provided instructions to the patient for basal insulin dose self-titration. Default instructions advised patients to increase the dose of insulin glargine 300 U/mL (Gla-300) by 2 units every 4 days for fasting glucose greater than 130 mg/dl, provided no values were less than 80 mg/dl. These instructions could be amended by the discharge team or primary care provider. Other than Gla-300, no additional prescriptions were pended. In the DOS arm, the primary team was instructed to verify and complete the discharge order set, which was launched by the study team."
"The participants in the DOS received follow-up telephone calls by the study nurse to facilitate ongoing basal insulin dose titration and hospital follow-up."

5-ix) Describe use parameters

Describe use parameters (e.g., intended “doses” and optimal timing for use). Clarify what instructions or recommendations were given to the user, e.g., regarding timing, frequency, heaviness of use, if any, or was the intervention used ad libitum.

	1	2	3	4	5	
subitem not at all important	○	○	◉	○	○	essential

Clear selection

You're editing your response. Sharing this URL allows others to also edit your response. FILL OUT A NEW RESPONSE

Does your paper address subitem 5-ix?

Copy and paste relevant sections from the manuscript (include quotes in quotation marks "like this" to indicate direct quotes from your manuscript), or elaborate on this item by providing additional information not in the ms, or briefly explain why the item is not applicable/relevant for your study

The DOS was a one-time use at the point of discharge.
"These instructions could be amended by the discharge team or primary care provider. In the DOS arm, the primary team was instructed to verify and complete the discharge order set, which was launched by the study team."

5-x) Clarify the level of human involvement

Clarify the level of human involvement (care providers or health professionals, also technical assistance) in the e-intervention or as co-intervention (detail number and expertise of professionals involved, if any, as well as “type of assistance offered, the timing and frequency of the support, how it is initiated, and the medium by which the assistance is delivered”. It may be necessary to distinguish between the level of human involvement required for the trial, and the level of human involvement required for a routine application outside of a RCT setting (discuss under item 21 – generalizability).

	1	2	3	4	5	
subitem not at all important	○	○	○	◉	○	essential

Clear selection

You're editing your response. Sharing this URL allows others to also edit your response.

FILL OUT A NEW RESPONSE

Does your paper address subitem 5-x?

Copy and paste relevant sections from the manuscript (include quotes in quotation marks "like this" to indicate direct quotes from your manuscript), or elaborate on this item by providing additional information not in the ms, or briefly explain why the item is not applicable/relevant for your study

"These instructions could be amended by the discharge team or primary care provider. Other than Gla-300, no additional prescriptions were pended. In the DOS arm, the primary team was instructed to verify and complete the discharge order set, which was pre-launched by the study team."

5-xi) Report any prompts/reminders used

Report any prompts/reminders used: Clarify if there were prompts (letters, emails, phone calls, SMS) to use the application, what triggered them, frequency etc. It may be necessary to distinguish between the level of prompts/reminders required for the trial, and the level of prompts/reminders for a routine application outside of a RCT setting (discuss under item 21 – generalizability).

	1	2	3	4	5	
subitem not at all important	○	○	○	○	◉	essential

Clear selection

Does your paper address subitem 5-xi? *

Copy and paste relevant sections from the manuscript (include quotes in quotation marks "like this" to indicate direct quotes from your manuscript), or elaborate on this item by providing additional information not in the ms, or briefly explain why the item is not applicable/relevant for your study

"These instructions could be amended by the discharge team or primary care provider. In the DOS arm, the primary team was instructed to verify and complete the discharge order

You're editing your response. Sharing this URL allows others to also edit your response.

FILL OUT A NEW RESPONSE

5-xii) Describe any co-interventions (incl. training/support)

Describe any co-interventions (incl. training/support): Clearly state any interventions that are provided in addition to the targeted eHealth intervention, as ehealth intervention may not be designed as stand-alone intervention. This includes training sessions and support [1]. It may be necessary to distinguish between the level of training required for the trial, and the level of training for a routine application outside of a RCT setting (discuss under item 21 – generalizability.

	1	2	3	4	5	
subitem not at all important	○	○	○	○	◉	essential

Clear selection

Does your paper address subitem 5-xii? *

Copy and paste relevant sections from the manuscript (include quotes in quotation marks "like this" to indicate direct quotes from your manuscript), or elaborate on this item by providing additional information not in the ms, or briefly explain why the item is not applicable/relevant for your study

"These instructions could be amended by the discharge team or primary care provider. In the DOS arm, the primary team was instructed to verify and complete the discharge order set, which was launched by the study team."
"The discharge order set also provided instructions to the patient for basal insulin dose self-titration. Default instructions advised patients to increase the dose of insulin glargine 300 U/mL (Gla-300) by 2 units every 4 days for fasting glucose greater than 130 mg/dl, provided no values were less than 80 mg/dl. "
"All participants in both treatment groups received a phone call at 2 weeks and 6 weeks following discharge in which data related to ambulatory and inpatient encounters, glucose monitoring and insulin use were collected."

You're editing your response. Sharing this URL allows others to also edit your response. FILL OUT A NEW RESPONSE

6a) Completely defined pre-specified primary and secondary outcome measures, including how and when they were assessed

Does your paper address CONSORT subitem 6a? *

Copy and paste relevant sections from the manuscript (include quotes in quotation marks "like this" to indicate direct quotes from your manuscript), or elaborate on this item by providing additional information not in the ms, or briefly explain why the item is not applicable/relevant for your study

"The primary outcome was the change in HbA1c from baseline to 24 weeks post-discharge." "Secondary outcomes related to hospital discharge included proportion of patients with new prescriptions for insulin and related supplies and clear discharge patient ordersinstructions, including correct frequency, no jargon or technical terms, correct quantity dispensed and refills using the following definitions:...".
"Post-discharge, self-reported insulin dosing and hypoglycemia were solicited. Secondary outcomes included HbA1c at 12 weeks, fasting glucose at 12 and 24 weeks, and proportion of subjects achieving HbA1c <7% (53 mmol/mol), < 6.5% (48 mmol/mol), or individualized HbA1c target, defined using Healthcare Effectiveness Data and Information Set (HEDIS) criteria.[21]"

6a-i) Online questionnaires: describe if they were validated for online use and apply CHERRIES items to describe how the questionnaires were designed/deployed

If outcomes were obtained through online questionnaires, describe if they were validated for online use and apply CHERRIES items to describe how the questionnaires were designed/deployed [9].

	1	2	3	4	5	
subitem not at all important	(●)	( )	( )	( )	( )	essential

You're editing your response. Sharing this URL allows others to also edit your response. FILL OUT A NEW RESPONSE

Does your paper address subitem 6a-i?

Copy and paste relevant sections from manuscript text

No questionnaires were used for outcomes.

6a-ii) Describe whether and how “use” (including intensity of use/dosage) was defined/measured/monitored

Describe whether and how “use” (including intensity of use/dosage) was defined/measured/monitored (logins, logfile analysis, etc.). Use/adoption metrics are important process outcomes that should be reported in any ehealth trial.

	1	2	3	4	5	
subitem not at all important	○	○	◉	○	○	essential

Clear selection

Does your paper address subitem 6a-ii?

Copy and paste relevant sections from manuscript text

"Secondary outcomes related to hospital discharge included proportion of patients with new prescriptions for insulin and related supplies and clear discharge patient ordersinstructions, including correct frequency, no jargon or technical terms, correct quantity dispensed and refills using the following definitions:"
""Finally, while the DOS increased the completeness of diabetes medications at discharge, it was launched by the study team in order to understand its utility under optimal use. Thus additional study of its usability, acceptability and implementation is needed."

You're editing your response. Sharing this URL allows others to also edit your response. FILL OUT A NEW RESPONSE

6a-iii) Describe whether, how, and when qualitative feedback from participants was obtained

Describe whether, how, and when qualitative feedback from participants was obtained (e.g., through emails, feedback forms, interviews, focus groups).

	1	2	3	4	5	
subitem not at all important	(•)	( )	( )	( )	( )	essential

Clear selection

Does your paper address subitem 6a-iii?

Copy and paste relevant sections from manuscript text

Qualitative feedback was not obtained.

6b) Any changes to trial outcomes after the trial commenced, with reasons

Does your paper address CONSORT subitem 6b? *

Copy and paste relevant sections from the manuscript (include quotes in quotation marks "like this" to indicate direct quotes from your manuscript), or elaborate on this item by providing additional information not in the ms, or briefly explain why the item is not applicable/relevant for your study

There were no modifications to trial outcomes after commencement.

You're editing your response. Sharing this URL allows others to also edit your response. FILL OUT A NEW RESPONSE

7a) How sample size was determined

NPT: When applicable, details of whether and how the clustering by care provides or centers was addressed

7a-i) Describe whether and how expected attrition was taken into account when calculating the sample size

Describe whether and how expected attrition was taken into account when calculating the sample size.

	1	2	3	4	5	
subitem not at all important	○	○	○	○	◉	essential

Clear selection

Does your paper address subitem 7a-i?

Copy and paste relevant sections from manuscript title (include quotes in quotation marks "like this" to indicate direct quotes from your manuscript), or elaborate on this item by providing additional information not in the ms, or briefly explain why the item is not applicable/relevant for your study

"We estimated a sample size of 111 individuals per group to achieve 80% power to detect a treatment difference of 0.8% in HbA1c adjusting for baseline clinical factors (age, insulin dose at discharge, and whether or not the patient was new to insulin), assuming 20% attrition, SD= 2.2% (standard deviation of HbA1c), r= 0.25 (correlation between baseline 24 week HbA1c) and R2 = 0. 5 (squared correlation between baseline factors and the outcome).[20]"

You're editing your response. Sharing this URL allows others to also edit your response.

FILL OUT A NEW RESPONSE

7b) When applicable, explanation of any interim analyses and stopping guidelines

Does your paper address CONSORT subitem 7b? *

Copy and paste relevant sections from the manuscript (include quotes in quotation marks "like this" to indicate direct quotes from your manuscript), or elaborate on this item by providing additional information not in the ms, or briefly explain why the item is not applicable/relevant for your study

There were no interim analyses or stopping guidelines identified a priori.

8a) Method used to generate the random allocation sequence

NPT: When applicable, how care providers were allocated to each trial group

Does your paper address CONSORT subitem 8a? *

Copy and paste relevant sections from the manuscript (include quotes in quotation marks "like this" to indicate direct quotes from your manuscript), or elaborate on this item by providing additional information not in the ms, or briefly explain why the item is not applicable/relevant for your study

"Randomization to the DOS or ESC was performed 1:1 using a random number generator program within the electronic data capture system (REDCap) and was stratified by admission insulin therapy."

8b) Type of randomisation; details of any restriction (such as blocking and block size)

You're editing your response. Sharing this URL allows others to also edit your response.

FILL OUT A NEW RESPONSE

Does your paper address CONSORT subitem 8b? *

Copy and paste relevant sections from the manuscript (include quotes in quotation marks "like this" to indicate direct quotes from your manuscript), or elaborate on this item by providing additional information not in the ms, or briefly explain why the item is not applicable/relevant for your study

"Randomization to the DOS or ESC was performed 1:1 using a random number generator program within the electronic data capture system (REDCap) and was stratified by admission insulin therapy."

9) Mechanism used to implement the random allocation sequence (such as sequentially numbered containers), describing any steps taken to conceal the sequence until interventions were assigned

Does your paper address CONSORT subitem 9? *

Copy and paste relevant sections from the manuscript (include quotes in quotation marks "like this" to indicate direct quotes from your manuscript), or elaborate on this item by providing additional information not in the ms, or briefly explain why the item is not applicable/relevant for your study

Randomization was concealed using REDCap. "Randomization to the DOS or ESC was performed 1:1 using a random number generator program within the electronic data capture system (REDCap) and was stratified by admission insulin therapy."

10) Who generated the random allocation sequence, who enrolled participants, and who assigned participants to interventions

You're editing your response. Sharing this URL allows others to also edit your response.

FILL OUT A NEW RESPONSE

Does your paper address CONSORT subitem 10? *

Copy and paste relevant sections from the manuscript (include quotes in quotation marks "like this" to indicate direct quotes from your manuscript), or elaborate on this item by providing additional information not in the ms, or briefly explain why the item is not applicable/relevant for your study

"Randomization to the DOS or ESC was performed 1:1 using a random number generator program within the electronic data capture system (REDCap) ..."
"Permission was obtained from the attending physician of the inpatient service prior to approaching the patient in person by the study coordinator for consent. "

11a) If done, who was blinded after assignment to interventions (for example, participants, care providers, those assessing outcomes) and how

NPT: Whether or not administering co-interventions were blinded to group assignment

11a-i) Specify who was blinded, and who wasn’t

Specify who was blinded, and who wasn’t. Usually, in web-based trials it is not possible to blind the participants [1, 3] (this should be clearly acknowledged), but it may be possible to blind outcome assessors, those doing data analysis or those administering co-interventions (if any).

	1	2	3	4	5	
subitem not at all important	○	○	○	○	◉	essential

Clear selection

You're editing your response. Sharing this URL allows others to also edit your response. FILL OUT A NEW RESPONSE

Does your paper address subitem 11a-i? *

Copy and paste relevant sections from the manuscript (include quotes in quotation marks "like this" to indicate direct quotes from your manuscript), or elaborate on this item by providing additional information not in the ms, or briefly explain why the item is not applicable/relevant for your study

The study was not blinded.

11a-ii) Discuss e.g., whether participants knew which intervention was the “intervention of interest” and which one was the “comparator”

Informed consent procedures (4a-ii) can create biases and certain expectations - discuss e.g., whether participants knew which intervention was the “intervention of interest” and which one was the “comparator”.

	1	2	3	4	5	
subitem not at all important	○	○	○	○	◉	essential

Clear selection

Does your paper address subitem 11a-ii?

Copy and paste relevant sections from the manuscript (include quotes in quotation marks "like this" to indicate direct quotes from your manuscript), or elaborate on this item by providing additional information not in the ms, or briefly explain why the item is not applicable/relevant for your study

The study was not blinded.

You're editing your response. Sharing this URL allows others to also edit your response. FILL OUT A NEW RESPONSE

11b) If relevant, description of the similarity of interventions

(this item is usually not relevant for ehealth trials as it refers to similarity of a placebo or sham intervention to a active medication/intervention)

You're editing your response. Sharing this URL allows others to also edit your response.

FILL OUT A NEW RESPONSE

Does your paper address CONSORT subitem 11b? *

Copy and paste relevant sections from the manuscript (include quotes in quotation marks "like this" to indicate direct quotes from your manuscript), or elaborate on this item by providing additional information not in the ms, or briefly explain why the item is not applicable/relevant for your study

"All participants in both treatment groups received a phone call at 2 weeks and 6 weeks following discharge in which data related to ambulatory and inpatient encounters, glucose monitoring and basal insulin use were collected. Basal insulin adherence was defined as >80% of doses taken in the previous week. The participants in the DOS received follow-up telephone calls by the study nurse to facilitate ongoing basal insulin dose titration and hospital follow-up. The nurse had a basic understanding of diabetes but was not a Certified Diabetes Care and Education Specialist. In the ESC group, follow-up telephone calls and visits were conducted on the same schedule as the ESC group but were conducted by the study coordinator for the purposes of information gathering only. In-person visits at 12 weeks and 24 weeks were conducted by the coordinator in the ESC group and by the nurse with or without the coordinator in the DOS group. At in-person visits, HbA1c and point-of care glucose were collected in addition to data at telephone visits."
"Background therapy/procedures
All participants received Gla-300 (provided at no cost to the participant) plus additional background therapy (non-insulin and prandial insulin therapies), as part of standard care determined by the hospital discharge team. A basic description of Gla-300 was provided to hospital teams using a standard template, recommending that Gla-300 be provided in 1 or 2 injections per day at the same time of day (only pens with 1 unit dosing increments were available). A 1:1 initial dosing conversion from in-hospital administration of glargine U100 or detemir to outpatient Gla-300 was recommended. The Diabetes Consult Service provided input only when requested by the primary service.
All patients received standard discharge instructions using the EMR, which features medication reconciliation, prescription generation, disease specific instructions, and follow-up appointments. Hospital discharge was coordinated by the primary team and case manager who arranged follow-up and any additional needs such as transportation prior to discharge. A discharge summary is sent to the primary care provider of record per routine practice. All patients were instructed to maintain a standardized study diary that records glucose levels and insulin doses as well as any hypoglycemic events and associated symptoms.""

You're editing your response. Sharing this URL allows others to also edit your response.

FILL OUT A NEW RESPONSE

12a) Statistical methods used to compare groups for primary and secondary outcomes

NPT: When applicable, details of whether and how the clustering by care providers or centers was addressed

Does your paper address CONSORT subitem 12a? *

Copy and paste relevant sections from the manuscript (include quotes in quotation marks "like this" to indicate direct quotes from your manuscript), or elaborate on this item by providing additional information not in the ms, or briefly explain why the item is not applicable/relevant for your study

"Follow-up data, including the primary and secondary outcomes, were analyzed using generalized linear mixed models. Continuous outcomes were analyzed using linear mixed models assuming an unstructured covariance matrix for the residual errors, and binary outcomes were analyzed using logistic regression models containing random subject specific intercepts. For some binary outcomes, we could not fit mixed models due to small cell counts. In these cases, data were analyzed cross-sectionally using separate logistic regression models fit to the data at each time point or Fisher's exact test, depending on the number of events. All models were adjusted for potential confounders, which we defined as any factors measured at baseline related to the outcome ($p < 0.1$) and that differed by a meaningful amount across treatment arms (difference in proportions of 10% or more, difference in means of 0.5 standard deviations or more) at any follow-up visit (Supplemental Table 2). In the primary analysis (change in HbA1c), we also adjusted for risk factors identified a priori (age, insulin dose at discharge, whether or not the patient was new to insulin) to increase the precision of our treatment effect estimate. In secondary analyses, Holm's method was used to account for multiple comparisons performed at 2, 6, 12, and 24 weeks.[22] Differences in components ordered at discharge were analyzed using Fisher's Exact Test."

You're editing your response. Sharing this URL allows others to also edit your response.

FILL OUT A NEW RESPONSE

12a-i) Imputation techniques to deal with attrition / missing values

Imputation techniques to deal with attrition / missing values: Not all participants will use the intervention/comparator as intended and attrition is typically high in ehealth trials. Specify how participants who did not use the application or dropped out from the trial were treated in the statistical analysis (a complete case analysis is strongly discouraged, and simple imputation techniques such as LOCF may also be problematic [4]).

	1	2	3	4	5	
subitem not at all important	○	○	○	○	●	essential

Clear selection

Does your paper address subitem 12a-i? *

Copy and paste relevant sections from the manuscript (include quotes in quotation marks "like this" to indicate direct quotes from your manuscript), or elaborate on this item by providing additional information not in the ms, or briefly explain why the item is not applicable/relevant for your study

"All outcomes were assessed according to the originally assigned group."
"HbA1c levels and healthcare utilization data were collected at study visits or extracted from the EMR when available in subjects with otherwise missing data."
However it is recognized that the level of drop-out in the primary outcome can not be addressed by imputation as the target sample size was not achieved. This is acknowledged in the abstract and the discussion. However, there was an adequate sample size to assess discharge prescriptions.

You're editing your response. Sharing this URL allows others to also edit your response.

FILL OUT A NEW RESPONSE

12b) Methods for additional analyses, such as subgroup analyses and adjusted analyses

Does your paper address CONSORT subitem 12b? *

Copy and paste relevant sections from the manuscript (include quotes in quotation marks "like this" to indicate direct quotes from your manuscript), or elaborate on this item by providing additional information not in the ms, or briefly explain why the item is not applicable/relevant for your study

"Follow-up data, including the primary and secondary outcomes, were analyzed using generalized linear mixed models. Continuous outcomes were analyzed using linear mixed models assuming an unstructured covariance matrix for the residual errors, and binary outcomes were analyzed using logistic regression models containing random subjectspecific intercepts. For some binary outcomes, we could not fit mixed models due to small cell counts. In these cases, data were analyzed cross-sectionally using separate logistic regression models fit to the data at each time point or Fisher's exact test, depending on the number of events. All models were adjusted for potential confounders, which we defined as any factors measured at baseline related to the outcome ($p < 0.1$) and that differed by a meaningful amount across treatment arms (difference in proportions of 10% or more, difference in means of 0.5 standard deviations or more) at any follow-up visit (Supplemental Table 2). In the primary analysis (change in HbA1c), we also adjusted for risk factors identified a priori (age, insulin dose at discharge, whether or not the patient was new to insulin) to increase the precision of our treatment effect estimate. In secondary analyses, Holm's method was used to account for multiple comparisons performed at 2, 6, 12, and 24 weeks.[22] Differences in components ordered at discharge were analyzed using Fisher's Exact Test."

X26) REB/IRB Approval and Ethical Considerations [recommended as subheading under "Methods"] (not a CONSORT item)

You're editing your response. Sharing this URL allows others to also edit your response.

FILL OUT A NEW RESPONSE

X26-i) Comment on ethics committee approval

	1	2	3	4	5	
subitem not at all important	○	○	○	○	◉	essential

Clear selection

Does your paper address subitem X26-i?

Copy and paste relevant sections from the manuscript (include quotes in quotation marks "like this" to indicate direct quotes from your manuscript), or elaborate on this item by providing additional information not in the ms, or briefly explain why the item is not applicable/relevant for your study

"This study was approved by the OSU Ohio State University Institutional Review Board and all patients signed informed consent." A separate subheading was not added because it was only 1 sentence.

x26-ii) Outline informed consent procedures

Outline informed consent procedures e.g., if consent was obtained offline or online (how? Checkbox, etc.?), and what information was provided (see 4a-ii). See [6] for some items to be included in informed consent documents.

	1	2	3	4	5	
subitem not at all important	○	○	◉	○	○	essential

Clear selection

You're editing your response. Sharing this URL allows others to also edit your response.

FILL OUT A NEW RESPONSE

Does your paper address subitem X26-ii?

Copy and paste relevant sections from the manuscript (include quotes in quotation marks "like this" to indicate direct quotes from your manuscript), or elaborate on this item by providing additional information not in the ms, or briefly explain why the item is not applicable/relevant for your study

"Permission was obtained from the attending physician of the inpatient service prior to approaching the patient in person by the study coordinator for consent."

X26-iii) Safety and security procedures

Safety and security procedures, incl. privacy considerations, and any steps taken to reduce the likelihood or detection of harm (e.g., education and training, availability of a hotline)

	1	2	3	4	5	
subitem not at all important	○	○	◉	○	○	essential

Clear selection

Does your paper address subitem X26-iii?

Copy and paste relevant sections from the manuscript (include quotes in quotation marks "like this" to indicate direct quotes from your manuscript), or elaborate on this item by providing additional information not in the ms, or briefly explain why the item is not applicable/relevant for your study

The patient signed consent contains information related to HIPAA but because there is no patient facing electronic component, this does not seem to apply. The institution uses Epic and has vigorous safety and security measures in place as a medical center.

You're editing your response. Sharing this URL allows others to also edit your response. FILL OUT A NEW RESPONSE

RESULTS

13a) For each group, the numbers of participants who were randomly assigned, received intended treatment, and were analysed for the primary outcome

NPT: The number of care providers or centers performing the intervention in each group and the number of patients treated by each care provider in each center

Does your paper address CONSORT subitem 13a? *

Copy and paste relevant sections from the manuscript (include quotes in quotation marks "like this" to indicate direct quotes from your manuscript), or elaborate on this item by providing additional information not in the ms, or briefly explain why the item is not applicable/relevant for your study

"A total of 158 patients signed consent (Supplemental Figure 1). Three patients did not receive the study intervention due to withdrawal of consent (2 subjects, 1 in each study arm) or no longer qualifying due to switch to U500 insulin prior to discharge (1, DOS arm)."
"Discharge data were available in 75 participants in the ESC arm and 80 participants in the DOS arm. "
"HbA1c was available in 54 subjects in each arm at 12 weeks, and 44 and 45 subjects in the DOS and ESC arms, respectively at 24 weeks."

13b) For each group, losses and exclusions after randomisation, together with reasons

You're editing your response. Sharing this URL allows others to also edit your response.

FILL OUT A NEW RESPONSE

Does your paper address CONSORT subitem 13b? (NOTE: Preferably, this is shown in a CONSORT flow diagram) *

Copy and paste relevant sections from the manuscript (include quotes in quotation marks "like this" to indicate direct quotes from your manuscript), or elaborate on this item by providing additional information not in the ms, or briefly explain why the item is not applicable/relevant for your study

"Three patients did not receive the study intervention due to withdrawal of consent (2 subjects, 1 in each study arm) or no longer qualifying due to switch to U500 insulin prior to discharge (1, DOS arm)."
"HbA1c was available in 54 subjects in each arm at 12 weeks, and 44 and 45 subjects in the DOS and ESC arms, respectively at 24 weeks. The remainder of participants were lost to follow-up."
"Discharge data were available in 75 participants in the ESC arm and 80 participants in the DOS arm."

13b-i) Attrition diagram

Strongly recommended: An attrition diagram (e.g., proportion of participants still logging in or using the intervention/comparator in each group plotted over time, similar to a survival curve) or other figures or tables demonstrating usage/dose/engagement.

	1	2	3	4	5	
subitem not at all important	(•)	( )	( )	( )	( )	essential

Clear selection

You're editing your response. Sharing this URL allows others to also edit your response. FILL OUT A NEW RESPONSE

Does your paper address subitem 13b-i?

Copy and paste relevant sections from the manuscript or cite the figure number if applicable (include quotes in quotation marks "like this" to indicate direct quotes from your manuscript), or elaborate on this item by providing additional information not in the ms, or briefly explain why the item is not applicable/relevant for your study

The order set is used only one time at discharge and therefore the attrition diagram does not apply.

14a) Dates defining the periods of recruitment and follow-up

Does your paper address CONSORT subitem 14a? *

Copy and paste relevant sections from the manuscript (include quotes in quotation marks "like this" to indicate direct quotes from your manuscript), or elaborate on this item by providing additional information not in the ms, or briefly explain why the item is not applicable/relevant for your study

"were enrolled between 5/1/2018-3/4/2020"

14a-i) Indicate if critical “secular events” fell into the study period

Indicate if critical “secular events” fell into the study period, e.g., significant changes in Internet resources available or “changes in computer hardware or Internet delivery resources”

	1	2	3	4	5	
subitem not at all important	○	○	○	◉	○	essential

Clear selection

You're editing your response. Sharing this URL allows others to also edit your response. FILL OUT A NEW RESPONSE

Does your paper address subitem 14a-i?

Copy and paste relevant sections from the manuscript (include quotes in quotation marks "like this" to indicate direct quotes from your manuscript), or elaborate on this item by providing additional information not in the ms, or briefly explain why the item is not applicable/relevant for your study

"No modifications were made to the DOS during the study. " The electronic medical record undergoes regular updates with periodic enhancements but none that would have significantly impacted the DOS.

14b) Why the trial ended or was stopped (early)

Does your paper address CONSORT subitem 14b? *

Copy and paste relevant sections from the manuscript (include quotes in quotation marks "like this" to indicate direct quotes from your manuscript), or elaborate on this item by providing additional information not in the ms, or briefly explain why the item is not applicable/relevant for your study

"However, study enrollment halted in March 2020 with the COVID-19 crisis and concerns with the feasibility of continuing to enroll and conduct study visits."

15) A table showing baseline demographic and clinical characteristics for each group

NPT: When applicable, a description of care providers (case volume, qualification, expertise, etc.) and centers (volume) in each group

You're editing your response. Sharing this URL allows others to also edit your response. FILL OUT A NEW RESPONSE

Does your paper address CONSORT subitem 15? *

Copy and paste relevant sections from the manuscript (include quotes in quotation marks "like this" to indicate direct quotes from your manuscript), or elaborate on this item by providing additional information not in the ms, or briefly explain why the item is not applicable/relevant for your study

"Baseline characteristics in each group are shown in Table 1. "

15-i) Report demographics associated with digital divide issues

In ehealth trials it is particularly important to report demographics associated with digital divide issues, such as age, education, gender, social-economic status, computer/Internet/ehealth literacy of the participants, if known.

	1	2	3	4	5	
subitem not at all important	○	○	◉	○	○	essential

Clear selection

Does your paper address subitem 15-i? *

Copy and paste relevant sections from the manuscript (include quotes in quotation marks "like this" to indicate direct quotes from your manuscript), or elaborate on this item by providing additional information not in the ms, or briefly explain why the item is not applicable/relevant for your study

The DOS is directed at clinicians and therefore ehealth literacy was not assessed in patients. However, other demographics including education level, employment, insurance, home ownership were collected and appear in Table 1.

You're editing your response. Sharing this URL allows others to also edit your response.

FILL OUT A NEW RESPONSE

16) For each group, number of participants (denominator) included in each analysis and whether the analysis was by original assigned groups

16-i) Report multiple “denominators” and provide definitions

Report multiple “denominators” and provide definitions: Report N’s (and effect sizes) “across a range of study participation [and use] thresholds” [1], e.g., N exposed, N consented, N used more than x times, N used more than y weeks, N participants “used” the intervention/comparator at specific pre-defined time points of interest (in absolute and relative numbers per group). Always clearly define “use” of the intervention.

	1	2	3	4	5	
subitem not at all important	○	○	○	○	◉	essential

Clear selection

Does your paper address subitem 16-i? *

Copy and paste relevant sections from the manuscript (include quotes in quotation marks "like this" to indicate direct quotes from your manuscript), or elaborate on this item by providing additional information not in the ms, or briefly explain why the item is not applicable/relevant for your study

"All outcomes were assessed according to the originally assigned group. "
"HbA1c levels and healthcare utilization data were collected at study visits or extracted from the EMR when available in subjects with otherwise missing data."
"HbA1c was available in 54 subjects in each arm at 12 weeks, and 44 and 45 subjects in the DOS and ESC arms, respectively at 24 weeks."
"Discharge data were available in 75 participants in the ESC arm and 80 participants in the DOS arm."

You're editing your response. Sharing this URL allows others to also edit your response.
FILL OUT A NEW RESPONSE

16-ii) Primary analysis should be intent-to-treat

Primary analysis should be intent-to-treat, secondary analyses could include comparing only “users”, with the appropriate caveats that this is no longer a randomized sample (see 18-i).

	1	2	3	4	5	
subitem not at all important	○	○	○	○	◉	essential

Clear selection

Does your paper address subitem 16-ii?

Copy and paste relevant sections from the manuscript (include quotes in quotation marks "like this" to indicate direct quotes from your manuscript), or elaborate on this item by providing additional information not in the ms, or briefly explain why the item is not applicable/relevant for your study

"All outcomes were assessed according to the originally assigned group. "

17a) For each primary and secondary outcome, results for each group, and the estimated effect size and its precision (such as 95% confidence interval)

You're editing your response. Sharing this URL allows others to also edit your response.

FILL OUT A NEW RESPONSE

Does your paper address CONSORT subitem 17a? *

Copy and paste relevant sections from the manuscript (include quotes in quotation marks "like this" to indicate direct quotes from your manuscript), or elaborate on this item by providing additional information not in the ms, or briefly explain why the item is not applicable/relevant for your study

"Patients in the DOS arm were more likely to receive new prescriptions for bolus insulin (21 [81%] vs 12 [44%]; P=0.01), needles/syringes (18 [95%] vs 15 [63%]; P=0.03), and glucometers (24 [86%] vs 9 [36%]; P=<0.001). "

17a-i) Presentation of process outcomes such as metrics of use and intensity of use

In addition to primary/secondary (clinical) outcomes, the presentation of process outcomes such as metrics of use and intensity of use (dose, exposure) and their operational definitions is critical. This does not only refer to metrics of attrition (13-b) (often a binary variable), but also to more continuous exposure metrics such as "average session length". These must be accompanied by a technical description how a metric like a "session" is defined (e.g., timeout after idle time) [1] (report under item 6a).

	1	2	3	4	5	
subitem not at all important	○	○	○	◉	○	essential

Clear selection

You're editing your response. Sharing this URL allows others to also edit your response.

FILL OUT A NEW RESPONSE

Does your paper address subitem 17a-i?

Copy and paste relevant sections from the manuscript (include quotes in quotation marks "like this" to indicate direct quotes from your manuscript), or elaborate on this item by providing additional information not in the ms, or briefly explain why the item is not applicable/relevant for your study

Patients in the DOS arm were more likely to receive new prescriptions for bolus insulin (21 [81%] vs 12 [44%]; P=0.01), needles/syringes (18 [95%] vs 15 [63%]; P=0.03), and glucometers (24 [86%] vs 9 [36%]; P=0.0002). During hospitalization, most participants reported sufficient home supplies of bolus insulin (65%), basal insulin (65%), lancets (52%), and a glucometer (66%). Needles and syringes however were insufficient in 93% of patients and test strips were insufficient in 57% (there were no continuous or intermittently scanned glucose monitors).
Process measures related to the DOS otherwise were not collected because the DOS was launched into the discharge navigator by the study team.
"Finally, while the DOS increased the completeness of diabetes medications at discharge, it was launched by the study team in order to understand its utility under optimal use. Thus, additional study of its usability, acceptability and implementation is needed."

17b) For binary outcomes, presentation of both absolute and relative effect sizes is recommended

You're editing your response. Sharing this URL allows others to also edit your response.

FILL OUT A NEW RESPONSE

Does your paper address CONSORT subitem 17b? *

Copy and paste relevant sections from the manuscript (include quotes in quotation marks "like this" to indicate direct quotes from your manuscript), or elaborate on this item by providing additional information not in the ms, or briefly explain why the item is not applicable/relevant for your study

"Patients in the DOS arm were more likely to receive new prescriptions for bolus insulin (21 [81%] vs 12 [44%]; P=0.01), needles/syringes (18 [95%] vs 15 [63%]; P=0.03), and glucometers (24 [86%] vs 9 [36%]; P=<0.00102). "
"Overall, errors or lack of clarity in discharge orders were similar between arms (44 [55%] in DOS vs. 51 [68%] in ESC). "
"Participants in the DOS arm were more likely to achieve an HbA1c below their HEDIS target at 12 weeks (22 [41%] vs 9 [17%]; OR 3.29, 95% confidence interval [CI] 1.32, 8.13], P=0.01); although this association did not persist to 24 weeks (16 [36%] vs 9 [20%]; OR 2.10, 95% CI 0.80, 5.55, P=0.13). "

18) Results of any other analyses performed, including subgroup analyses and adjusted analyses, distinguishing pre-specified from exploratory

You're editing your response. Sharing this URL allows others to also edit your response.

FILL OUT A NEW RESPONSE

Does your paper address CONSORT subitem 18? *

Copy and paste relevant sections from the manuscript (include quotes in quotation marks "like this" to indicate direct quotes from your manuscript), or elaborate on this item by providing additional information not in the ms, or briefly explain why the item is not applicable/relevant for your study

"All secondary outcomes were pre-specified except for hospital readmission which was considered to be an exploratory outcome."
"Readmissions by 30 days (exploratory outcome) occurred in 17 (13%) of subjects overall. "

18-i) Subgroup analysis of comparing only users

A subgroup analysis of comparing only users is not uncommon in ehealth trials, but if done, it must be stressed that this is a self-selected sample and no longer an unbiased sample from a randomized trial (see 16-iii).

	1	2	3	4	5	
subitem not at all important	○	○	○	◉	○	essential

Clear selection

You're editing your response. Sharing this URL allows others to also edit your response.

FILL OUT A NEW RESPONSE

Does your paper address subitem 18-i?

Copy and paste relevant sections from the manuscript (include quotes in quotation marks "like this" to indicate direct quotes from your manuscript), or elaborate on this item by providing additional information not in the ms, or briefly explain why the item is not applicable/relevant for your study

"Finally, while the DOS increased the completeness of diabetes medications at discharge, it was launched by the study team in order to understand its utility under optimal use. Thus additional study of its usability, acceptability, and implementation is needed."

19) All important harms or unintended effects in each group

(for specific guidance see CONSORT for harms)

Does your paper address CONSORT subitem 19? *

Copy and paste relevant sections from the manuscript (include quotes in quotation marks "like this" to indicate direct quotes from your manuscript), or elaborate on this item by providing additional information not in the ms, or briefly explain why the item is not applicable/relevant for your study

"Ironically, one artifact may have been introduced by the multi-faceted intervention itself, which required a switch in basal insulin at discharge, often late in the hospitalization and possibly after other discharge orders were populated."

You're editing your response. Sharing this URL allows others to also edit your response. FILL OUT A NEW RESPONSE

19-i) Include privacy breaches, technical problems

Include privacy breaches, technical problems. This does not only include physical "harm" to participants, but also incidents such as perceived or real privacy breaches [1], technical problems, and other unexpected/unintended incidents. "Unintended effects" also includes unintended positive effects [2].

	1	2	3	4	5	
subitem not at all important	○	◉	○	○	○	essential

Clear selection

Does your paper address subitem 19-i?

Copy and paste relevant sections from the manuscript (include quotes in quotation marks "like this" to indicate direct quotes from your manuscript), or elaborate on this item by providing additional information not in the ms, or briefly explain why the item is not applicable/relevant for your study

Because there is no patient facing electronic component, this does not seem to apply. The institution uses Epic and has vigorous safety and security measures in place as a medical center.

You're editing your response. Sharing this URL allows others to also edit your response.

FILL OUT A NEW RESPONSE

19-ii) Include qualitative feedback from participants or observations from staff/researchers

Include qualitative feedback from participants or observations from staff/researchers, if available, on strengths and shortcomings of the application, especially if they point to unintended/unexpected effects or uses. This includes (if available) reasons for why people did or did not use the application as intended by the developers.

	1	2	3	4	5	
subitem not at all important	○	○	○	◉	○	essential

Clear selection

Does your paper address subitem 19-ii?

Copy and paste relevant sections from the manuscript (include quotes in quotation marks "like this" to indicate direct quotes from your manuscript), or elaborate on this item by providing additional information not in the ms, or briefly explain why the item is not applicable/relevant for your study

"However there are opportunities to improve the clarity of insulin instructions in the EMR. , as Dosing fields are typically inadequate, resulting in a need to use free text fields or provide discharge instructions via a separate workflow, which is external to the electronic medication reconciliation process [11]. Additional customization could include fields that account for oral intake, or glucose level, or time of day, guide patient self-titration, or calculate quantity dispensed. In particular, decision support could provide guidance for converting flexible meal dosing (the standard practice at the study institution) to fixed meal dosing, which is more appropriate for many patients. Additional benefit could be achieved by enhancing remote monitoring programs post-discharge and device (smart insulin pen and/or glucose monitor) integration within the EMR. Ironically, one artifact may have been introduced by the multi-faceted intervention itself, which required a switch in basal insulin at discharge, often late in the hospitalization and possibly after other discharge orders were populated. Following closure of the study and after obtaining feedback from the institution's multidisciplinary inpatient Glucose Management Committee, additional [illegible]

You're editing your response. Sharing this URL allows others to also edit your response. FILL OUT A NEW RESPONSE

DISCUSSION

22) Interpretation consistent with results, balancing benefits and harms, and considering other relevant evidence

NPT: In addition, take into account the choice of the comparator, lack of or partial blinding, and unequal expertise of care providers or centers in each group

22-i) Restate study questions and summarize the answers suggested by the data, starting with primary outcomes and process outcomes (use)

Restate study questions and summarize the answers suggested by the data, starting with primary outcomes and process outcomes (use).

	1	2	3	4	5	
subitem not at all important	○	○	○	○	◉	essential

Clear selection

You're editing your response. Sharing this URL allows others to also edit your response. FILL OUT A NEW RESPONSE

Does your paper address subitem 22-i? *

Copy and paste relevant sections from the manuscript (include quotes in quotation marks "like this" to indicate direct quotes from your manuscript), or elaborate on this item by providing additional information not in the ms, or briefly explain why the item is not applicable/relevant for your study

"In this study, an insulin-specific DOS was developed to address barriers to prescribing insulin and to improve the hospital transition of care among persons with T2D. The DOS resulted in improvements in new prescriptions for bolus insulin, needles, and testing supplies, but did not improve order clarity. Follow-up data were available in a subset of patients, for whom there were favorable trends in adjusted HbA1c, despite early discontinuation of study recruitment. These data from the first study to implement a dedicated discharge order set among hospitalized patients with T2D requiring insulin provide important insights for optimizing hospital diabetes discharge programs."

22-ii) Highlight unanswered new questions, suggest future research

Highlight unanswered new questions, suggest future research.

	1	2	3	4	5	
subitem not at all important	○	○	○	○	◉	essential

Clear selection

You're editing your response. Sharing this URL allows others to also edit your response.

FILL OUT A NEW RESPONSE

Does your paper address subitem 22-ii?

Copy and paste relevant sections from the manuscript (include quotes in quotation marks "like this" to indicate direct quotes from your manuscript), or elaborate on this item by providing additional information not in the ms, or briefly explain why the item is not applicable/relevant for your study

"Future iterations should also consider optimization of non-insulin therapies, particularly to reduce cardiorenal risk.[28]"
"However there are opportunities to improve the clarity of insulin instructions in the EMR. Dosing fields are typically inadequate, resulting in a need to use free text fields or provide discharge instructions via a separate workflow, which is external to the electronic medication reconciliation process [11]. Additional customization could include fields that account for oral intake, glucose level, or time of day, guide patient self-titration, or calculate quantity dispensed. In particular, decision support could provide guidance for converting flexible meal dosing (the standard practice at the study institution) to fixed meal dosing, which is more appropriate for many patients. Additional benefit could be achieved by enhancing remote monitoring programs post-discharge and device (smart insulin pen and/or glucose monitor) integration within the EMR. Ironically, one artifact may have been introduced by the multi-faceted intervention itself, which required a switch in basal insulin at discharge, often late in the hospitalization and possibly after other discharge orders were populated. Following closure of the study and after obtaining feedback from the institution's multidisciplinary inpatient Glucose Management Committee, additional revisions to the DOS were made, including presenting pens as the preferred choice, and adding concentrated and premix insulin orders."

20) Trial limitations, addressing sources of potential bias, imprecision, and, if relevant, multiplicity of analyses

You're editing your response. Sharing this URL allows others to also edit your response.

FILL OUT A NEW RESPONSE

20-i) Typical limitations in ehealth trials

Typical limitations in ehealth trials: Participants in ehealth trials are rarely blinded. Ehealth trials often look at a multiplicity of outcomes, increasing risk for a Type I error. Discuss biases due to non-use of the intervention/usability issues, biases through informed consent procedures, unexpected events.

	1	2	3	4	5	
subitem not at all important	○	○	○	○	◉	essential

Clear selection

Does your paper address subitem 20-i? *

Copy and paste relevant sections from the manuscript (include quotes in quotation marks "like this" to indicate direct quotes from your manuscript), or elaborate on this item by providing additional information not in the ms, or briefly explain why the item is not applicable/relevant for your study

"As noted in the previous paragraph, limitations of the study relate to loss to follow-up, even within the context of a specifically dedicated study staff and the other enhancements. The COVID-19 pandemic also presented the largest barriers to carrying out study procedures, as drop-out was more common among patients who were enrolled in the 6 months prior to the start of the pandemic. Unfortunately, which we were only partially able to address this with virtual or minimal contact strategies; this highlights the overall vulnerability of our patient population. Furthermore, it is unknown whether similar results would be achieved in other populations (type 1 diabetes, non-insulin requiring, broader age range). As with other multi-component interventions, it is difficult to discern which components of the discharge instruction or the nursing support were the primary determinants of success. Finally, while the DOS increased the completeness of diabetes medications at discharge, it was launched by the study team in order to understand its utility under optimal use. Thus, additional study of its usability, acceptability and implementation is needed. For example, it would have been useful to quantify the amount of time saved through use of the order set, due to the presence of prefilled fields and decision support.

You're editing your response. Sharing this URL allows others to also edit your response. FILL OUT A NEW RESPONSE

21) Generalisability (external validity, applicability) of the trial findings

NPT: External validity of the trial findings according to the intervention, comparators, patients, and care providers or centers involved in the trial

21-i) Generalizability to other populations

Generalizability to other populations: In particular, discuss generalizability to a general Internet population, outside of a RCT setting, and general patient population, including applicability of the study results for other organizations

	1	2	3	4	5	
subitem not at all important	○	○	○	○	◉	essential

Clear selection

Does your paper address subitem 21-i?

Copy and paste relevant sections from the manuscript (include quotes in quotation marks "like this" to indicate direct quotes from your manuscript), or elaborate on this item by providing additional information not in the ms, or briefly explain why the item is not applicable/relevant for your study

"While the study population was generalizable due to the broad inclusion criteria, the limited follow-up data greatly impacts the ability to assess the external validity of the post-discharge component of the intervention."
"Furthermore, it is unknown whether similar results would be achieved in other populations (type 1 diabetes, non-insulin requiring, broader age range). "
"Finally, while the DOS increased the completeness of diabetes medications at discharge, it was launched by the study team in order to understand its utility under optimal use. Thus additional study of its usability, acceptability and implementation is needed."

You're editing your response. Sharing this URL allows others to also edit your response.

FILL OUT A NEW RESPONSE

21-ii) Discuss if there were elements in the RCT that would be different in a routine application setting

Discuss if there were elements in the RCT that would be different in a routine application setting (e.g., prompts/reminders, more human involvement, training sessions or other co-interventions) and what impact the omission of these elements could have on use, adoption, or outcomes if the intervention is applied outside of a RCT setting.

	1	2	3	4	5	
subitem not at all important	○	○	○	◉	○	essential

Clear selection

Does your paper address subitem 21-ii?

Copy and paste relevant sections from the manuscript (include quotes in quotation marks "like this" to indicate direct quotes from your manuscript), or elaborate on this item by providing additional information not in the ms, or briefly explain why the item is not applicable/relevant for your study

"Finally, while the DOS increased the completeness of diabetes medications at discharge, it was launched by the study team in order to understand its utility under optimal use. Thus additional study of its usability, acceptability and implementation is needed."

OTHER INFORMATION

23) Registration number and name of trial registry

You're editing your response. Sharing this URL allows others to also edit your response.

FILL OUT A NEW RESPONSE

Does your paper address CONSORT subitem 23? *

Copy and paste relevant sections from the manuscript (include quotes in quotation marks "like this" to indicate direct quotes from your manuscript), or elaborate on this item by providing additional information not in the ms, or briefly explain why the item is not applicable/relevant for your study

"Trial Registration: NCT03455985 Effectiveness of a Diabetes Focused Discharge Order Set Among Poorly Controlled Hospitalized Patients Transitioning to Glargine U300 Insulin"

24) Where the full trial protocol can be accessed, if available

Does your paper address CONSORT subitem 24? *

Cite a Multimedia Appendix, other reference, or copy and paste relevant sections from the manuscript (include quotes in quotation marks "like this" to indicate direct quotes from your manuscript), or elaborate on this item by providing additional information not in the ms, or briefly explain why the item is not applicable/relevant for your study

"Protocol: The full protocol is available upon request to the corresponding author."

25) Sources of funding and other support (such as supply of drugs), role of funders

You're editing your response. Sharing this URL allows others to also edit your response. FILL OUT A NEW RESPONSE

Does your paper address CONSORT subitem 25? *

Copy and paste relevant sections from the manuscript (include quotes in quotation marks "like this" to indicate direct quotes from your manuscript), or elaborate on this item by providing additional information not in the ms, or briefly explain why the item is not applicable/relevant for your study

"Funding source: This investigator sponsored study received funding from Sanofi."

X27) Conflicts of Interest (not a CONSORT item)

X27-i) State the relation of the study team towards the system being evaluated

In addition to the usual declaration of interests (financial or otherwise), also state the relation of the study team towards the system being evaluated, i.e., state if the authors/evaluators are distinct from or identical with the developers/sponsors of the intervention.

	1	2	3	4	5	
subitem not at all important	○	○	○	○	◉	essential

Clear selection

You're editing your response. Sharing this URL allows others to also edit your response.

FILL OUT A NEW RESPONSE

Does your paper address subitem X27-i?

Copy and paste relevant sections from the manuscript (include quotes in quotation marks "like this" to indicate direct quotes from your manuscript), or elaborate on this item by providing additional information not in the ms, or briefly explain why the item is not applicable/relevant for your study

"KD discloses research support from Novo Nordisk, Sanofi , Viacyte, Abbott, and Dexcom. consulting with Eli Lilly, Novo Nordisk, Boehringer-Ingelheim, Dexcom, and Tolerion, and honorarium from UptoDate, and Elsevier. MP discloses research support from Pfizer. EB discloses research support from Dexcom and Juvenile Diabetes Research Foundation. KW discloses research support: Sanofi & Allergan. Consulting: Novo; honorarium from Nova Biomedical. DB, CH, AS, JL and AW have no disclosures."

About the CONSORT EHEALTH checklist

As a result of using this checklist, did you make changes in your manuscript? *

- ○ yes, major changes
- ◉ yes, minor changes
- ○ no

What were the most important changes you made as a result of using this checklist?

We addressed process measures more clearly.

You're editing your response. Sharing this URL allows others to also edit your response. FILL OUT A NEW RESPONSE

How much time did you spend on going through the checklist INCLUDING making changes in your manuscript *

We spent approximately 7 hours going through the initial checklist and over 1 hour making the revisions for round one and 1 hour for round two.

As a result of using this checklist, do you think your manuscript has improved? *

- ○ yes
- ○ no
- ◉ Other: going through the checklist to update with revisions was tedious and [illegible]

Would you like to become involved in the CONSORT EHEALTH group?

This would involve for example becoming involved in participating in a workshop and writing an "Explanation and Elaboration" document

- ○ yes
- ◉ no
- ○ Other:

Clear selection

You're editing your response. Sharing this URL allows others to also edit your response. FILL OUT A NEW RESPONSE

Any other comments or questions on CONSORT EHEALTH

The amount of time is excessive; there should be a better way to save and come back to it.

STOP - Save this form as PDF before you click submit

To generate a record that you filled in this form, we recommend to generate a PDF of this page (on a Mac, simply select "print" and then select "print as PDF") before you submit it.

When you submit your (revised) paper to JMIR, please upload the PDF as supplementary file.

Don't worry if some text in the textboxes is cut off, as we still have the complete information in our database. Thank you!

Final step: Click submit !

Click submit so we have your answers in our database!

Submit

Never submit passwords through Google Forms.

This content is neither created nor endorsed by Google. Report Abuse - Terms of Service - Privacy Policy

Google Forms

You're editing your response. Sharing this URL allows others to also edit your response. FILL OUT A NEW RESPONSE
